# Assessment of ITER radiation environment during the remote-handling operation of In-Vessel components with D1SUNED

**DOI:** 10.1038/s41598-023-30534-x

**Published:** 2023-03-02

**Authors:** P. Martínez-Albertos, P. Sauvan, M. J. Loughlin, Y. Le Tonqueze, R. Juárez

**Affiliations:** 1 Dept. Ingeniería Energética, Universidad Nacional de Educación a Distancia (UNED), C/ Juan del Rosal 12, 28040 Madrid, Spain; 2 ITER Organization, Route de Vinon-sur-Verdon, CS 90 046, 13067 St. Paul Lez Durance Cedex, France; 3 Present Address: Oak Ridge National Laboratory, One Bethel Valley Road, Oak Ridge, TN USA

**Keywords:** Nuclear fusion and fission, Mechanical engineering, Software

## Abstract

During ITER operational life, a remote-handled cask will be used to transfer In-Vessel components to the Hot Cell for maintenance, storage and decommissioning purposes. Due to the distribution of penetrations for system allocation in the facility, the radiation field of each transfer operation presents a high spatial variability; all operations must be studied independently for workers and electronics protection. In this paper, we present a fully representative approach to describe the radiation environment during the complete remote-handling scenario of In-Vessel components in the ITER facility. The impact of all relevant radiation sources during different stages of the operation is addressed. As-built structures and 2020 baseline designs are considered to produce the most detailed neutronics model of the Tokamak Complex, the 400,000-tonne civil structure hosting the tokamak, up to date. Novel capabilities of the D1SUNED code have allowed to compute the integral dose, the dose rate and the photon-induced neutron flux of both moving and static radiation sources. Time bins are included in the simulations to compute the dose rate caused by In-Vessel components at all positions along the transfer. The time evolution of the dose rate is built in video format with a 1-m resolution, especially valuable for hot-spots identification.

## Introduction

ITER, the spearhead project in fusion power, aims to demonstrate the feasibility of nuclear fusion as a reliable energy source at a large scale. During its 500 MW pulse operation, around 1.77·10^20^ neutrons of 14.1 MeV will be produced every second, product of the deuterium–tritium fusion reactions. The intense neutron field will interact with the nearby materials (especially those of components from inside the vessel), transmuting and activating them. Such activated components entail a secondary and delayed gamma radiation source, which may be radiologically negligible compared to plasma neutrons during machine operation, but which become the main radiation source in the facility during machine shutdown.

**Figure 1:**
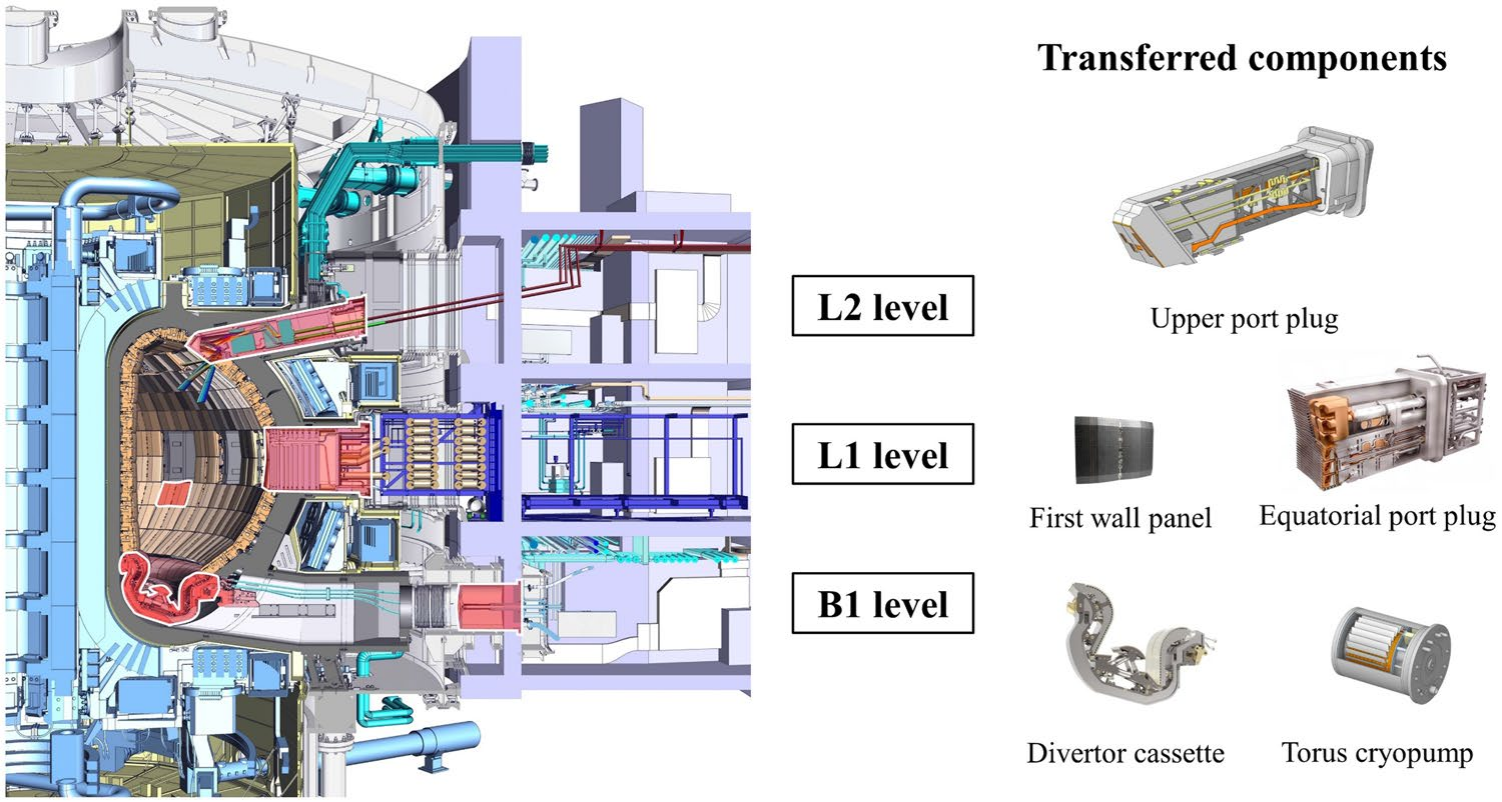
Cross-section of the ITER tokamak. In-Vessel components which are remotely transferred are shown and their locations within the tokamak are highlighted. The 3 levels of the tokamak are displayed.

During ITER’s operational life, it is anticipated the need of maintenance, storage, and decommissioning tasks of In-Vessel components, to be performed in the Hot Cell Complex. The 440 first wall panels, the 54 divertor cassettes and all port plugs, among other items (shown in Fig. 1), will be object of such tasks. But first, these components will need to be transferred to the Hot Cell from the Tokamak Complex. A remote-handled cask will be used to such purpose due to the high activation. The transfer operation comprises several stages, such as the removal of the bioshield plug, the loading of the component into the cask, the port cell door opening and the transfer itself. Consequently, alterations on the radiation field are expected as both the source and shielding geometries change in the facility during such operations. The assessment of the radiation field is required to check the compliance with the radiological zoning for workers protection and to support electronics qualification programs if necessary.

Previous works have addressed this issue^1^, however, new efforts are required due to (i) the need to follow an exhaustive approach regarding radiation sources and cask operations, (ii) the constant evolution of buildings and components designs, and (iii) the improvement of codes and methodologies.

The methodological capability of computing radiation maps due to moving radiation sources was proven by previous work^1^. Nonetheless, the remote-handling scenario of In-Vessel components could not be completely represented due to the following reasons:

- A limited set of cask trajectories were considered. Only 1 trajectory, starting from port cell #10, was considered for the upper port plug. Radiation maps of equatorial port plugs transfers were not addressed.
- Only one stage of the operation, the cask transfer to the Hot Cell, was studied. The radiation environment produced at other stages, such as the one present when opening the port cell door, were not addressed.

Also, Tokamak Complex designs have evolved and its construction has been nearly finished since previous work. The geometrical Monte Carlo N-Particle (MCNP)^2^ model previously used was simplistic (with barely any system penetrations in building walls) and is currently out-dated. As-built component structures and 2020 baseline designs have been considered in this study. Approximately 4800 penetrations of systems crossing building walls and slabs were included.

Finally, with regards to the methodology and code improvement, novel capabilities of the D1SUNED code^3^ have been developed. They have allowed not only to compute the integral dose caused by moving radiation sources, but also to discretize such dose within time bins. The result is the time evolution of the dose rate during the cask transfer in video format. This is especially valuable to identify compromising cask locations for design optimisation. Radiation maps due to photon-induced neutrons (or photoneutrons) from the first wall panels beryllium have been produced for the first time.

The work presented in this article has shed further light on the ITER radiation environment during the remote-handling operation scenario of In-Vessel components. The systematic approach to describe it, together with the updated Tokamak Complex geometry, the novel methodology employed, and some relevant results are explained in the following sections.

### Remote-handling scenario systematic approach

ITER transfer operation scenario is wide and complex. It involves various tasks and components, in addition of mixing both remote and hands-on controlled operations. In this study, only the remote-handling operation scenario of In-Vessel components has been addressed. It comprises the transfer of:

- 14 equatorial port plugs from diagnostics, test blanket modules and both electron and ion cyclotron heating systems.
- 14 upper port plugs from diagnostics and electron cyclotron heating systems.
- 54 divertor cassettes.
- 440 first-wall panels.
- 6 torus cryopumps, 6 in-vessel-viewing systems and 3 diagnostic racks.

**Figure 2:**
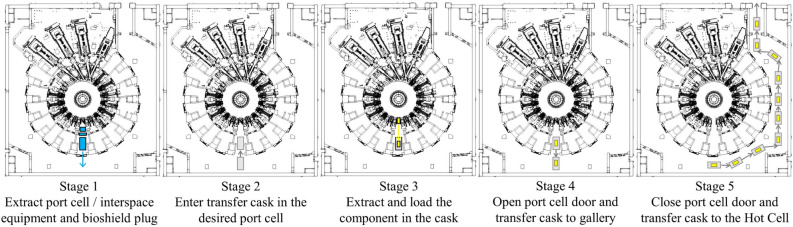
Simplified representation of the stages during the cask transfer operation from port cell #14. The transfer cask is shown in grey, the activated transferred component in yellow, the equipment to be removed beforehand in blue.

Transfer operations vary depending on the component considered and the port cell where they are being performed. Greatly simplifying the process, the transfer operation of a port plug comprises the following stages (see Fig. 2):

1. Extracting the port cell equipment. Extracting the bioshield plug and interspace equipment.
2. Opening the port cell door, entering transfer cask in the port cell and closing the port cell door.
3. Removing the port plug and loading it in transfer cask. This stage takes approximately 16 h.
4. Opening the port cell door and transferring the loaded cask to the gallery. This takes 30 min approximately.
5. Closing the port cell door and continuing the cask transfer to the Hot Cell. Depending on the port cell where the cask transfer begins, this stage may take from 1 to 6 h.

Among the stages previously mentioned, numbers 3, 4 and 5 present radiation fields which are particularly intense for this work. Those are:

- The radiation field produced by the activated component to be transferred.
- The radiation field produced by all the activated components remaining In-Vessel during the transfer.

Extraction operations of other components, such as the first wall panels or the divertor cassettes, may include more stages but the associated radiation fields remain the same. All components are transferred one by one, except the panels, three of which are loaded per cask. Port plugs, torus cryopump, in-vessel-viewing systems and racks are transferred from their corresponding port cell. First wall panels and divertor cassettes have, respectively, four and three port cells assigned to be extracted from.

In this study, a fully representative approach has been followed to describe the radiation environment of the remote-handling maintenance scenario of In-Vessel components in the Tokamak Complex. Five different components (shown in Fig. 1) have been considered as representative of all transferred items: (i) a divertor cassette, (ii) a torus cryopump, both at lower level (B1), (iii) 3 first wall panels and (iv) an equatorial port plug, at ground level (L1), and (v) an upper port plug at upper level (L2).

The selection of the specific port plugs and first wall panel models was based on a scoping analysis, attending to conservatism and maturity of the design. The model providing the highest dose-rate, but which had passed its Final Design Review and had explicit modelling of water channels in the first wall has been selected. The Ion-Cyclotron-Heating plug and the Electron-Cyclotron-Heating plug were the selected equatorial and upper port plugs respectively. Regarding the panels, the model of row #18, which presented explicit modelling of water lines, was considered.

In-vessel-viewing systems and diagnostics racks are represented with the cryopump. The activation of these 3 components is expected to be similar, and negligible compared to the activation of the divertor cassette.

In total, the study of the complete maintenance scenario was reduced to 41 operations from 37 different port cells. They correspond to (i) 14 upper port plug transfers at L2, (ii) 14 equatorial port plugs and 4 first wall panels transfers at L1, (iii) 3 divertor and 6 torus cryopump transfers at B1. Four port cells are shared by the panels and port plugs transfers at L1.

### Tokamak complex MCNP model

Since the previous study, two official MCNP models of the ITER Tokamak Complex have been released. The updates are associated to changes in the design and the availability of as-built geometries as the construction nears completion. The former model represented a step forward regarding the model quality^4^. The latter, described in this article, follows the same methodology but extends the applicability range and increases the accuracy by including several buildings, structures and components not considered in previous Tokamak Complex models.

The Tokamak Complex includes three buildings: The Tokamak Building (B11), hosting the machine, the Tritium Building (B14), where tritium will be processed, and the Diagnostic Building (B74), which will house the control and processing electronics of most of the diagnostic systems. As-built structure geometries have been considered for the update of B11. For those structures not yet built, 2020 baseline designs have been considered. B14 and B74 MCNP models have been recycled from previous model with minor modifications.

**Figure 3:**
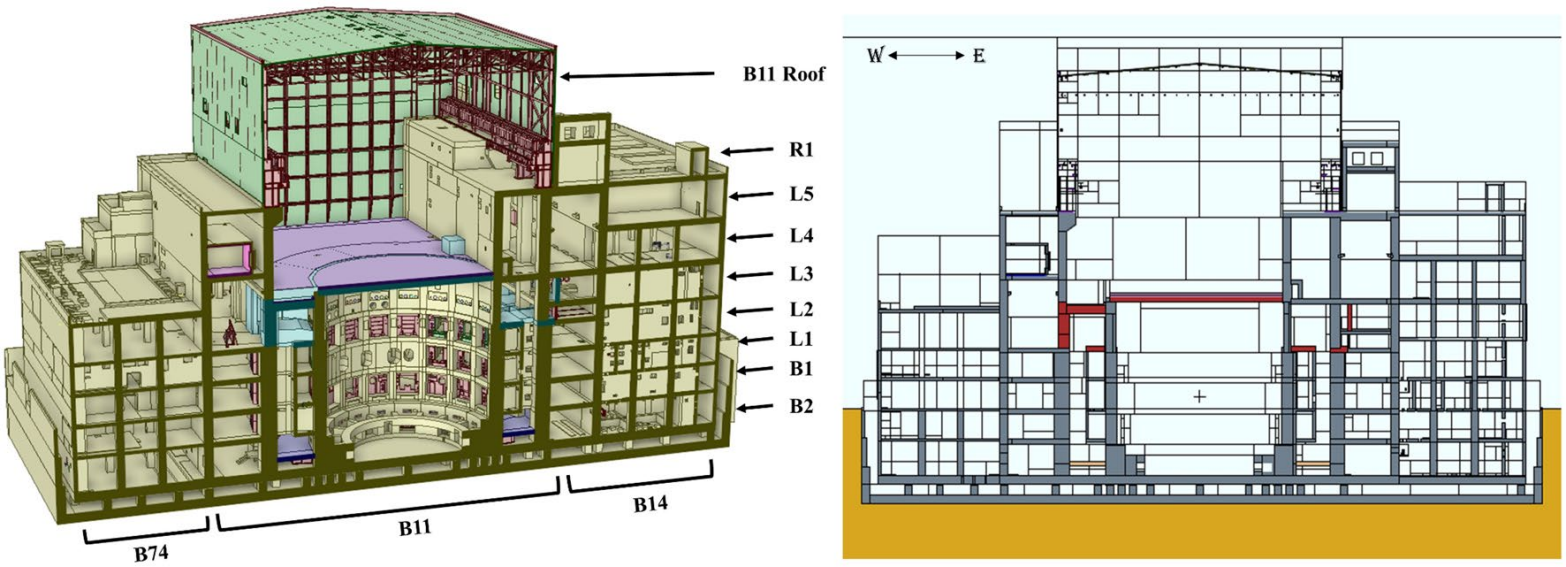
Cross section of the Tokamak Complex CAD model (left) and MCNP model (right). Buildings and levels are marked. Different colours in the MCNP model view indicate different materials.

Figure 3 shows a vertical cross-section of the CAD and MCNP models of the Tokamak Complex respectively. Levels are specified (B2 and B1 for underground levels, and from L1 to L5, plus R1, for those above the ground).

The facility comprises thousands of penetrations to accommodate the supporting systems, dedicated to machine control, plasma heating, diagnostics, cooling, fuelling, vacuum pump, cable trays, power supplies, heating, ventilation, and air conditioning, among others. Consequently, the Tokamak Complex radiation environment combines both attenuation and streaming phenomena and presents a high spatial variability. To consider such openings, all penetrations of systems crossing the building structures of B11, about 4800 in total, were modelled. Their locations, dimensions and materials have been updated. Dedicated backfilling cells (i.e., the component filling the gap between the wall/slab and the crossing system) are considered. Furthermore, the largest B14 and B74 penetrations were updated to the 2020 baseline design.

Another modification concerns the presence of 15 shielding measures designed after previous radiation maps. Aiming to reduce radiation levels in specific areas inside and outside the Tokamak Complex, they have been considered in the current model.

**Figure 4:**
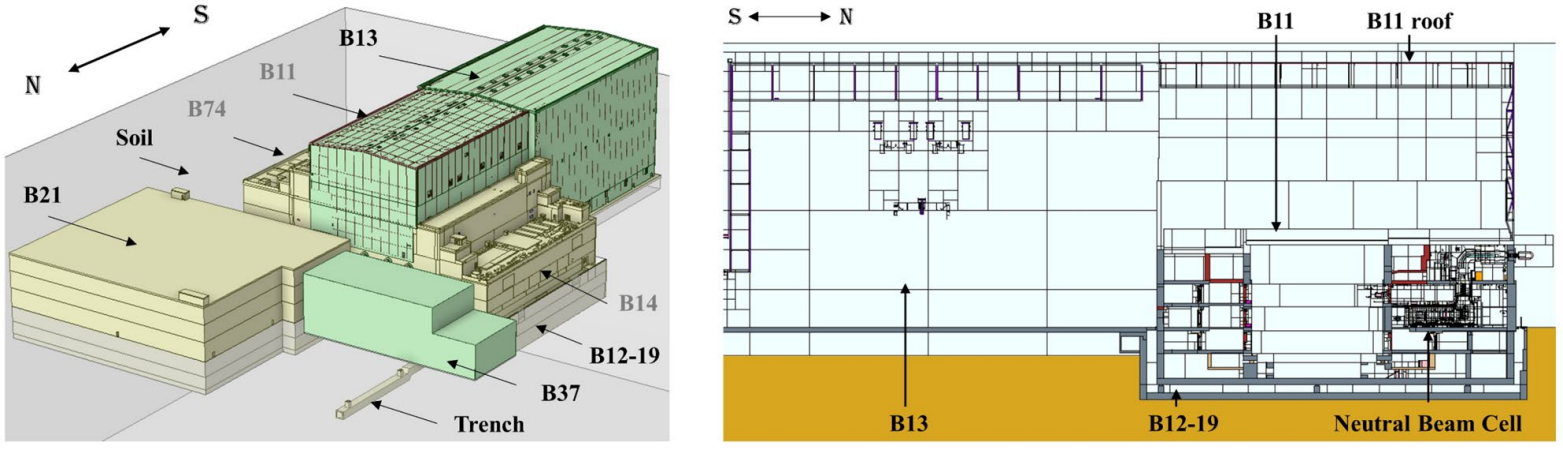
View of the adjacent buildings and the Tokamak Complex of CAD model (left) and MCNP model (right). Buildings and components are marked. The site north–south direction is shown in both figures.

Simplified geometries of the adjacent buildings have been integrated into the MCNP model. They include the B11 roof, the Seismic Platform (B12-19), the Assembly Hall (B13), the Hot Cell (B21), the High Voltage Building (B37) and the trench between B12-19 and B37. A simplified representation of the ITER soil has been considered, as well as air cells up to 1 km from the Tokamak Complex. A general view of the CAD and MCNP models are shown in Fig. 4.

Additionally, the MCNP model includes a detailed description of the Neutral Beam Cell and High Voltage Deck environment, and the Tokamak Cooling Water System^5^, which are out of the scope of this article.

### Computational considerations

**Figure 5:**
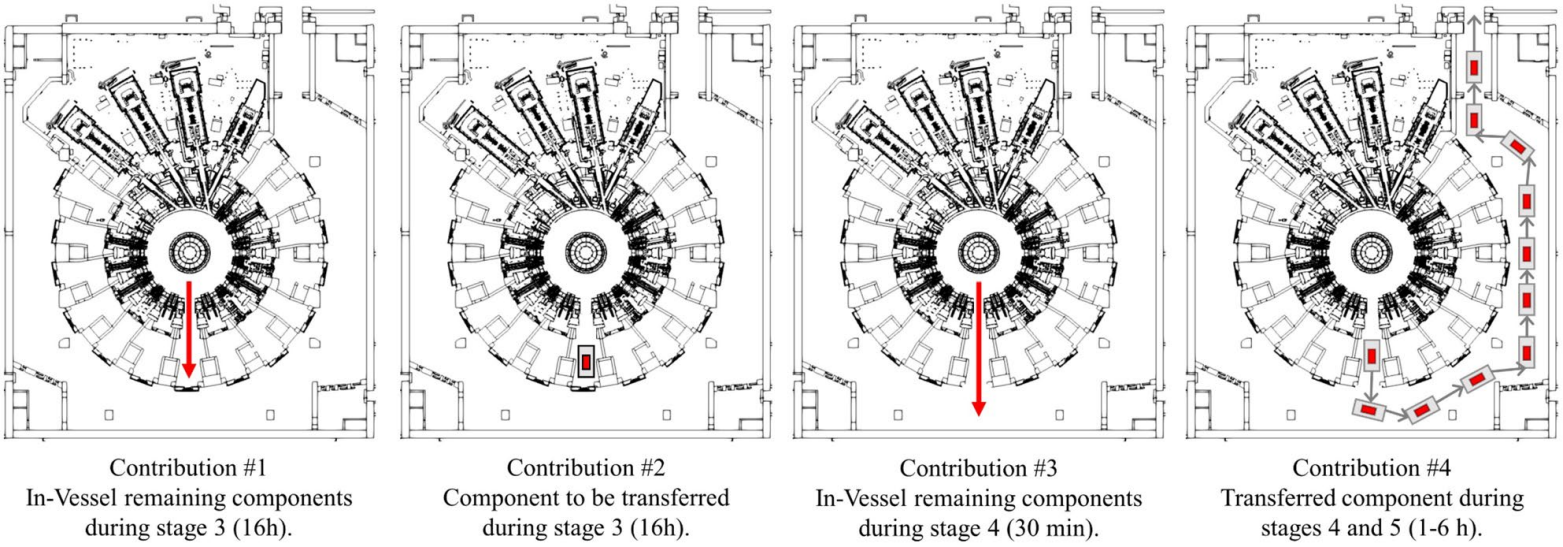
Computational breakdown of the radiation environment during the stages of a single transfer operation from port cell #14. Contributions to the radiation field are marked either with a red arrow (for components remaining In-Vessel) or red square (for transferred component). Images show the different Tokamak Complex geometries considered.

For computational convenience, the evaluation of the two radiation fields associated to the stages of a single cask transfer (Fig. 2) was decoupled into 4 contributions, summarised in Fig. 5. These are:

- Contribution #1. Due to the components remaining In-Vessel during stage 3 (loading the component into the cask).
- Contribution #2. Produced by the component to be transferred during stage 3 (loading the component into the cask).
- Contribution #3. Due to the components remaining In-Vessel during stage 4 (opening the port cell door and transfer the cask to the gallery).
- Contribution #4. Produced by the transferred component during stages 4 and 5 (opening the port cell door and transfer the cask to the gallery, closing the port cell door and continue the cask transfer to the Hot Cell).

Variations of the Tokamak Complex MCNP model have been prepared to represent the geometry of each stage. The interspace and port cell equipment, as well as the bioshield plug are removed in all contributions by changing their materials to air. The port plug has been also removed from its position for all contributions. The component to be transferred (either a port plug, a divertor cassette, the 3 first wall panels or a torus cryopump) has been placed, within the cask, inside the port cell during contribution #2. For contribution #4, the methodology used to deal with moving radiation sources is explained in the next section. The port cell door is open (air) for contributions #3 and #4, while it remains closed for contributions #1 and #2.

**Figure 6:**
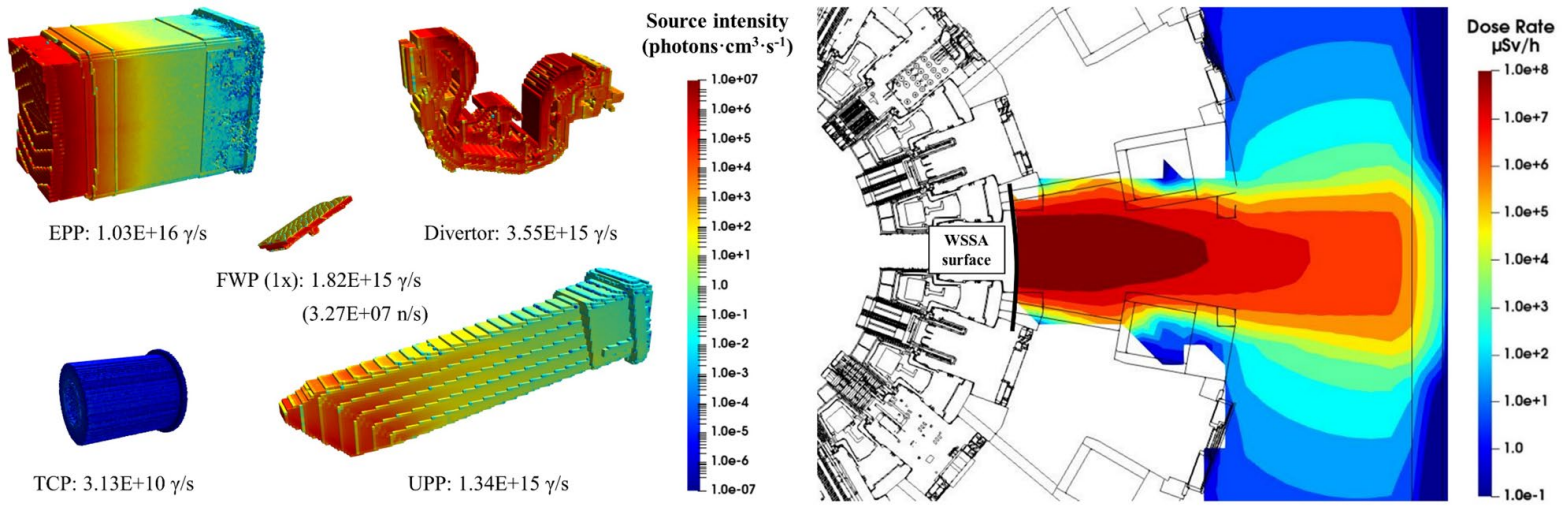
Computational breakdown of the radiation environment during the stages of a single transfer operation from port cell #14. Contributions to the radiation field are marked either with a red arrow (for components remaining In-Vessel) or red square (for transferred component). Images show the different Tokamak Complex geometries considered.

In this study, decay gamma radiation sources of In-Vessel components, produced by the exposure to the neutron fluence from the fusion reactions, have been considered. Additionally, the delayed photoneutron source, emerging when the beryllium present in the first wall panels is exposed to the panels decay gamma field, has also been addressed, as they may have an impact on components which would not be exposed to neutrons^6^. Such sources have been recorded using a geometry superimposed mesh with spatial resolution between 2 × 2 × 2 and 4 × 4 × 4 cm^3^, depending on the component. All sources and their intensities are shown in Fig. 6.

Decay gamma sources from the activated components remaining In-Vessel have been modelled using SRC-UNED^7^ methodology. This allows to link the information from the in-bioshield MCNP model, E-lite^8^, to the out-bioshield model, the Tokamak Complex MCNP model already described. To properly capture the machine configuration when this radiation source is relevant (stages 3 and 4), several modifications were implemented in E-lite. The interspace and port cell equipment, together with the bioshield plug and port plug, were removed from the geometry.

All radiation sources have been computed using the complete ITER lifetime irradiation scenario (SA2) followed by 3 weeks of cooling time. Cell-under-Voxel capability^9^ has been employed to only record information in cells of the desired components. The activation of the buildings has not been addressed in this study, as is expected to be negligible compared to the other radiation sources.

### About moving radiation sources and discretisation in time

**Figure 7:**
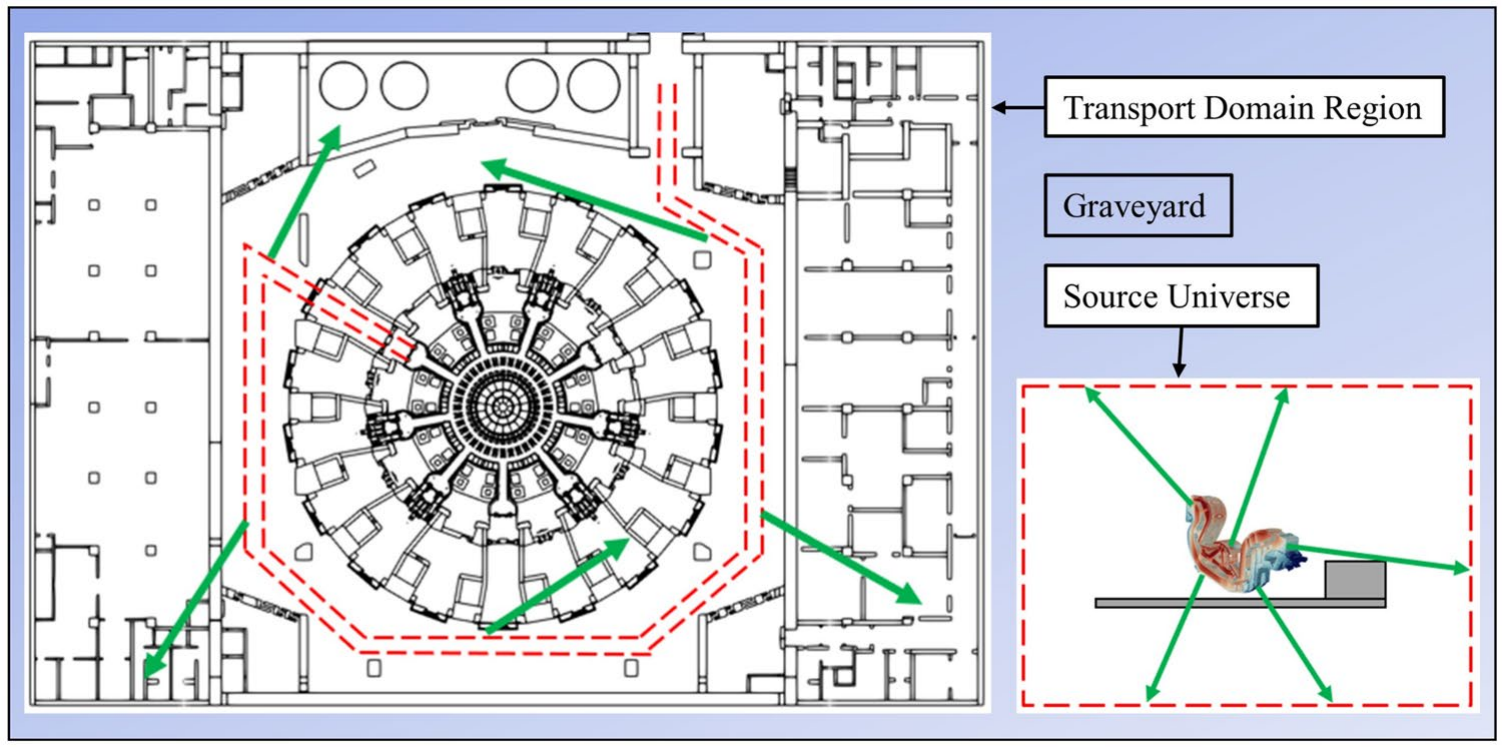
Geometry representation, not to scale, of the D1SUNED methodology for moving radiation sources. Boundaries of source universe and its trace along the transport domain region during its movement are dashed in red. Green lines represent decay photons.

To deal with calculations of moving radiation sources, new D1SUNED capabilities were developed. The methodology required to define two independent regions in the same MCNP input: the transport domain region and the source universe. In this study, the former is the already mentioned Tokamak Complex MCNP model. The latter includes the geometry of the transferred component and a simplified representation of the transfer cask. These two regions are separated by a graveyard (zone where radiation is not transported); thus, particles cannot travel through them in a normal simulation. A schematic view is shown in Fig. 7.

The cask trajectory must be supplied in a separate text file. The spatial resolution has been set by defining a sufficient number of points along a curve to have a smooth trajectory. For each point considered, the file contains the cask centroid coordinates, the cask angle with respect to a reference axis and the time.

Particles are initially sampled and transported on the source universe, according to the source distribution and geometry. Once they reach the boundaries of the source universe, they are sampled in the transport domain region according to the times defined in the trajectory file. The higher the time separating two points, the higher number of events are sampled. The second sampling does not alter the particles’ energy or direction. Finally, particles are transported as in normal MCNP simulations in the transport domain region.

This methodology is very similar to the one from the previous study^1^, as the same principles are assumed. However, the one proposed here brings a clear advantage: only one simulation is required to transport source particles in the Tokamak Complex model, and not two. This is computationally simpler and saves assumptions that must be made on the second simulation.

Computing radiation maps of static radiation sources is a straightforward task, but when dealing with moving radiation sources, the discretization in time of the desired nuclear quantity is required. D1SUNED v.4.1.1 allows to define time bins, in the same way the user would define spatial or energy bins in the mesh to tally the results. Now, the time evolution of nuclear quantities produced by moving radiation sources can be computed in a single calculation; there is no need to perform multiple simulations modifying the MCNP model geometry. Such novel capability has been applied to the cask transfer of In-Vessel components in ITER facility.

It is worth mentioning that the definition of a separated universe for the radiation source entails a natural consequence: the source geometry is not considered in the transport domain region. This causes the underestimation of quantities, tallied in the transport domain region, in those areas which would be inside the source universe if geometries were not independent (see the region inside the source universe, i.e. cask universe, from Fig. 9 or Fig. 12). Bear in mind that particles are sampled in the transport domain region once they reach the boundaries of the source universe, not before. A superimposed mesh defined in the former will not contain information of what is happening inside the latter. Note that this occurs, for a certain voxel, only during the time when the cask is “placed” in that same voxel. This issue is shared by all methodologies which separate the source geometry. In this study, this effect has been mitigated as much as possible by reducing the source universe size to a minimal extent.

## Results and discussion

**Figure 8:**
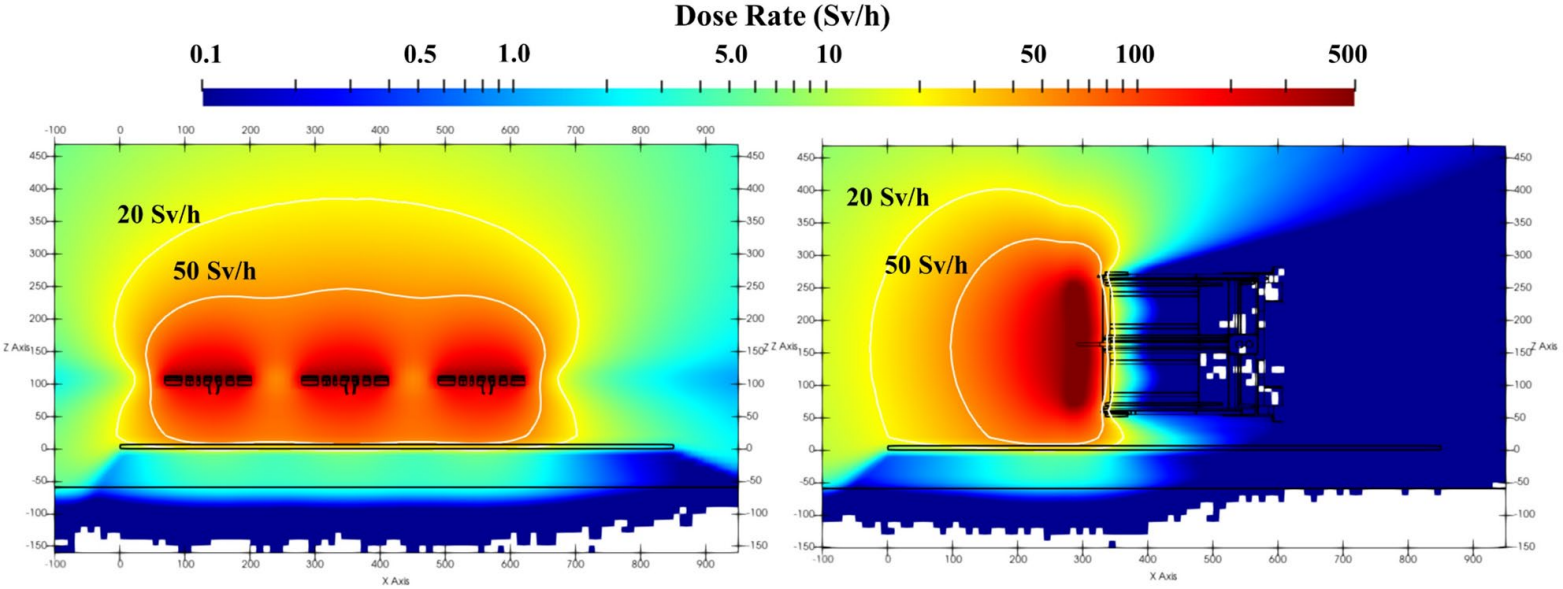
Vertical view of the dose rate (in Sv/h) for the 3 first wall panels and the equatorial port plug cask. The 20 and 50 Sv/h contour lines are shown.

Clearly, each radiation source influences results differently. However, this study has shown that both the transfer cask trajectory and the orientation of the component within the cask are key factors to be considered. Due to the high number of walls and slabs penetrations at the ITER facility, the variation on these factors makes certain streaming paths more likely than others. Figure 8 shows the different dose rate distributions within the cask for the first wall panels and the equatorial port plug.

The produced maps have been compared to previous results^1^ to the possible extent. Differences are observed but they may be explained by the use of different (and much more detailed, as previously mentioned) MCNP models of both the Tokamak Complex and the transferred components.

### Integral biological dose

Due to the computational breakdown described in previous sections, computing the integral dose of a certain cask operation requires combining the results from contributions shown in Fig. 5.

**Figure 9:**
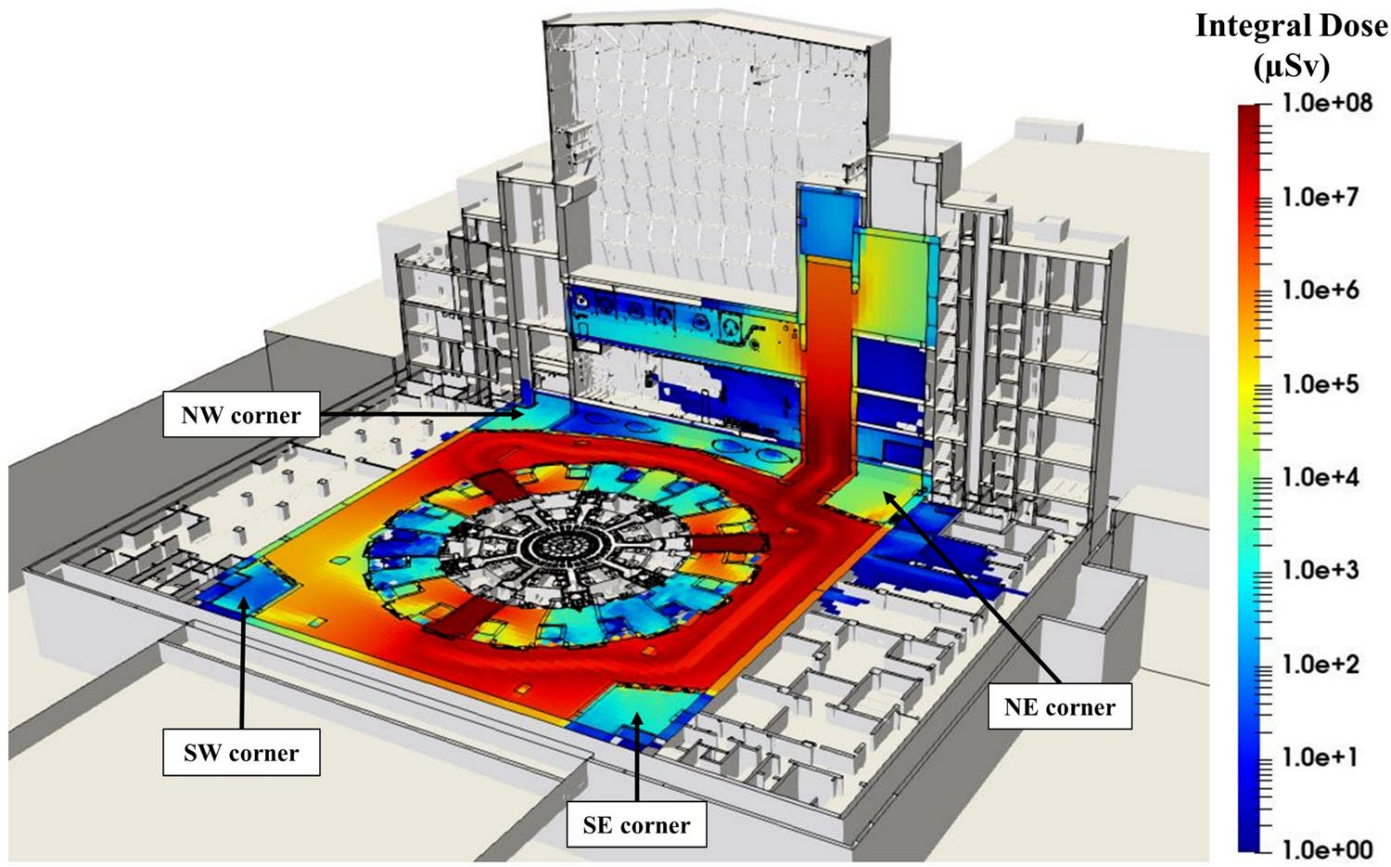
Integral total dose map (in μSv) produced by the extraction of the 54 divertor cassettes. Each of the 18 operations from port cells #2, #8 and #14, comprises contributions #2, #3 and #4 from Fig. 5. B1 level shielded corners are marked.

As an example, we consider the integral dose during the extraction of all divertors, shown in Fig. 9. Although there are 54 cassettes, they are only extracted from 3 port cells at B1 level: port cells #02, #08 and #14. The total integral dose would be the sum of the product of the integral dose of a cask operation from a single port cell, and the number of operations performed from that same port. In this case, 18 extractions were considered for each port cell. The integral dose of a single cask operation is the sum of the contributions computed for that port cell.

An analysis of the results has shown that the most important contributions outside the port cell are #3 and #4. Activated components remaining In-Vessel cause a non-negligible contribution during the time the port cell door is open (i.e., contribution #3). Depending on the region, this may be more relevant than the one produced by the component during its transfer (i.e., contribution #4); it cannot be neglected and must be studied thoroughly at all port cells.

Contribution #1 and #2 are only relevant in the port cell and negligible elsewhere, the former being smaller than the latter outside the port cell. For this reason, contribution #1 was not accounted for to generate the integral dose of a certain cask operation.

### Dose rate during the cask transfer

**Figure 10:**
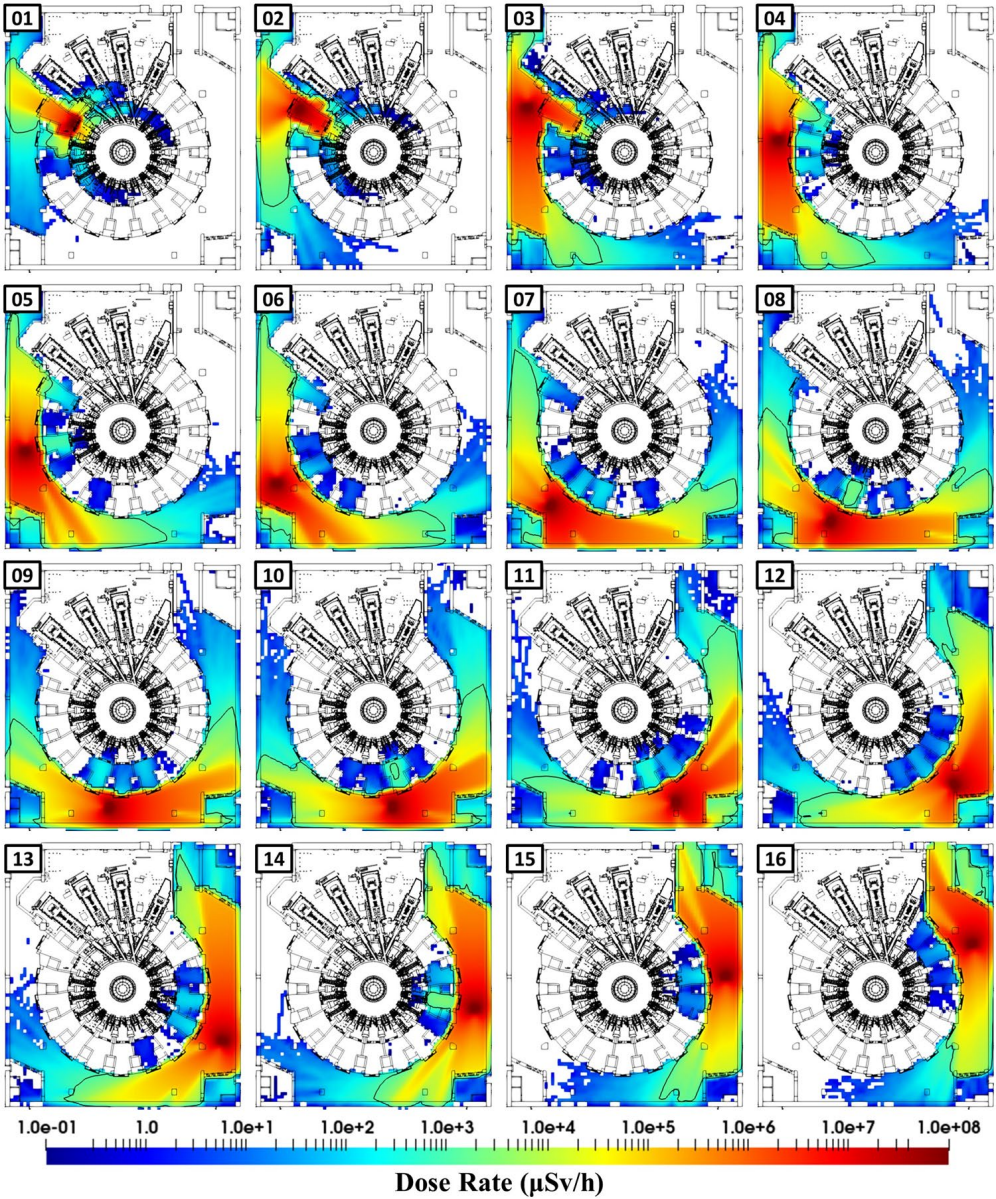
Time evolution of the dose rate maps (in μSv/h) produced by the transfer of the equatorial port plug cask from port cell #08 to the Hot Cell. The black line shows the 1 mSv/h dose rate contour. Dose rates below 0.1 μSv/h are not shown.

The dose rate produced by the activated component during its transfer (contribution 4 from Fig. 5), at all positions along its longest trajectory (i.e. from port cell #08 to the Hot Cell), has been computed. The dose rate time evolution in the facility has been produced in a video format (see the supplementary material). Each video frame corresponds to the dose rate averaged during the time it takes the cask to move 1 m. Figure 10 shows a small selection of the nearly 200 maps corresponding to the 1-m travelled distance over the 200 m of the trajectory from port cell #08 to the Hot Cell. The travelled distance by the cask from one image to the next one is 10 m approximately All nearly 200 maps have been computed in a single simulation where 1e11 sampled events have been considered.

**Figure 11:**
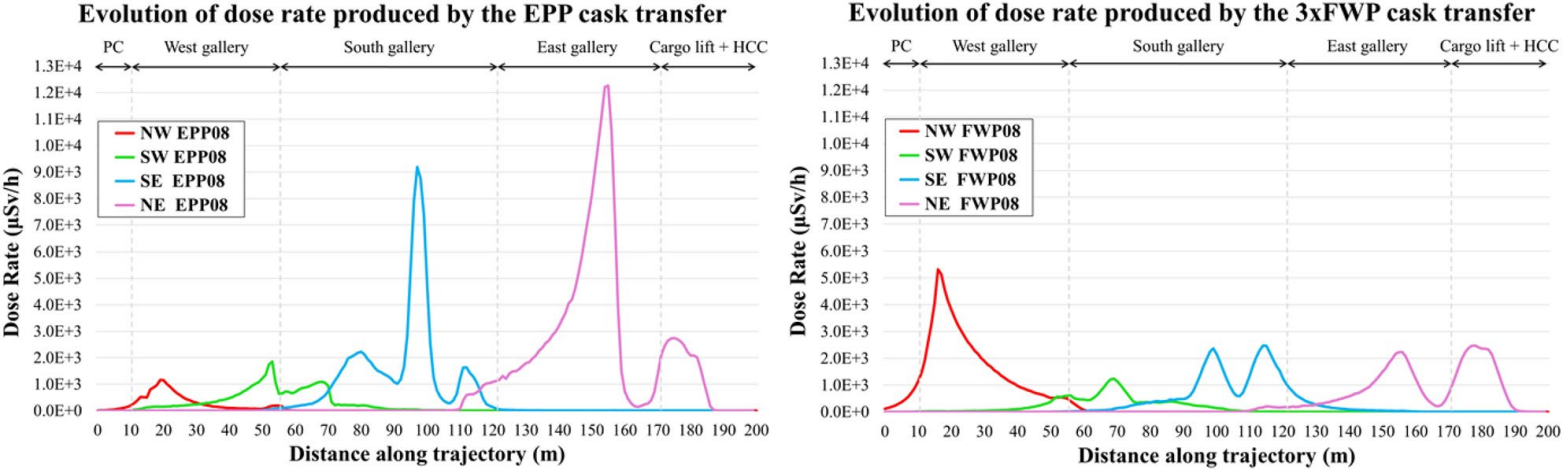
Time evolution of the dose rate, averaged on the L1 shielded corners, for the transfer of the equatorial port plug and 3 first wall panels over travelled distance. Positions within the port cell, west, south and east galleries and cargo lift and beyond are shown.

Figure 11 shows the complex nature of the radiation environment during the transfer of an equatorial port plug and first wall panels cask. It shows the dose rate, averaged over the northwest (NW), northeast (NE), southwest (SW) and southeast (SE) shielded corners of B11 at L1 level (B1 shielded corners are shown in Fig. 9) over the cask travelled distance.

Besides identifying the dose rate peaks and the cask positions associated to them, Fig. 11 demonstrates how the orientation of the component, and the dose rate distribution (see Fig. 8) associated to it, impact on results. As an example, at the NE corner, the port plug transfer from port cell #8 provides the highest dose rate. This is because the first wall of the port plug is facing north once it enters the east side of the gallery; it is “pointing” towards the NE corner. On the other hand, the dose rate at the NW corner is higher for the first wall panels transfer, as the first wall of the port plug is facing south once it exits port cell #8.

### Photoneutrons

**Figure 12:**
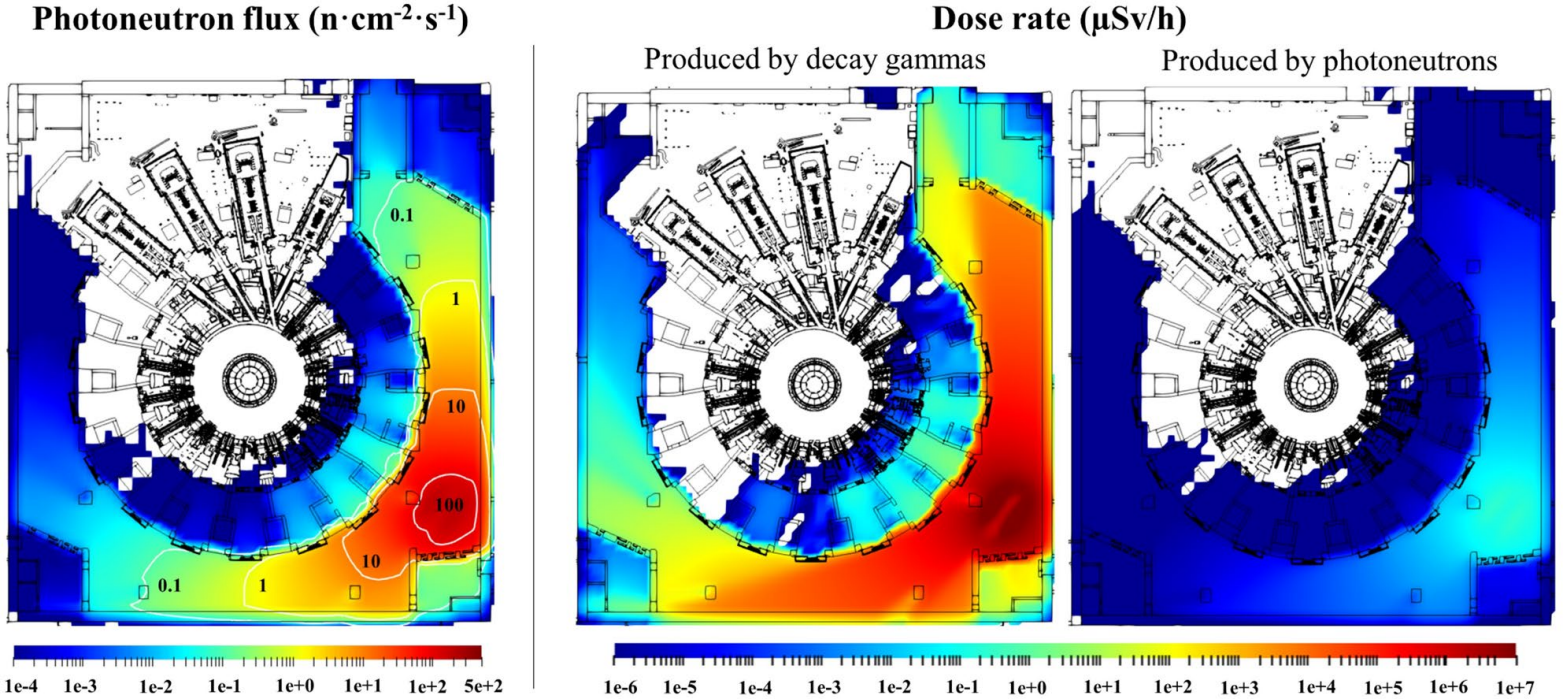
Radiation maps from the first wall panels cask in front of the L1 south-east shielded corner. Left: Photoneutron flux (in n·cm^−2^·s^−1^) and contour lines. Centre: Dose rate (in µSv/h) produced by decay gammas. Right: Dose rate (in µSv/h) produced by photoneutrons.

Radiation maps of photoneutrons emitted from the first wall panels beryllium have been produced in the Tokamak Complex for the first time. The cask, loaded with 3 panels, has been placed in different locations of the geometry. Figure 12 shows an example of the photoneutron flux in front of the SE shielded corner, where neutron flux should be below 10 n·cm^−2^·s^−1^ so critical electronics hosted there can operate under acceptable radiation conditions. Previous studies have addressed the compliance with such limit for plasma neutrons during operation of the machine^10^, but maps of beryllium photoneutrons during the transfer of the first wall panels were never produced. The photoneutron flux does not challenge the compliance of such limit. Figure 12 also shows the contribution of photoneutrons to the dose rate. As it can be seen, it is negligible with respect to the contribution of decay gammas from the first wall panels cask.

## Conclusions

The radiation environment in the ITER facility will change during the remote-handling maintenance scenario of In-Vessel components. Transfer cask operations require the extraction of highly activated components from all port cells and their movement through the galleries. To that end, the port cell door must open and the bioshield plug and other equipment should be removed beforehand. Such configuration creates a streaming phenomenon from inside the vessel to the port cell and beyond. Consequently, the radiation environment of each transfer operation presents a high spatial variability, and all operations must be studied thoroughly.

The ITER radiation environment during the remote-handling maintenance scenario of In-Vessel components has been addressed following a systematic approach regarding transfer operations and radiation sources. Most relevant contributions to the radiation environment have been considered, some for the first time, such as the one caused by the components remaining In-Vessel when the port cell door is open.

As-built geometries and 2020 baseline designs have been considered to update the MCNP model of the Tokamak Complex. Around 4800 system penetrations in building walls and slabs have been included. Simplified geometries of Auxiliary buildings, such as the Hot Cell, the Assembly Hall or the Seismic slab have also been considered. A detailed description of the Neutral Beam Cell and High Voltage Deck environment, and the Tokamak Cooling Water System has been included.

Novel capabilities of D1SUNED have been developed to compute, in a single simulation, nuclear quantities produced during the movement of radiation sources. Additionally, the time discretization during the cask movement has allowed to compute the evolution of the dose rate in a video format, especially valuable for design optimisation. Maps of photoneutron flux from first wall panels beryllium have been computed for the first time, and the compliance of electronics limits in the shielded corners has been checked.

The work presented in this article has improved the knowledge of ITER radiation environment during the remote-handling maintenance scenario of In-Vessel components. The high-quality results produced have been incorporated into the ITER official radiation maps set.

## Methods

### MCNP model and computational considerations

The simplification process of the Tokamak Complex CAD model was carried out using Space Claim^11^, while CAD to MCNP translation was performed with SuperMC^12,13^. Global Variance Reduction^14^ was used in the calculations. The number of events considered in the simulations, both in variance reduction and production runs, is in the range of 1e9 and 1e11. Statistical errors, shown in the supplementary material, are below 10% in the regions of interest, as recommended by MCNP. A multiplicative safety factor of 2 is applied to the results following the recommendations of ITER Organization.

### Radiation sources

All radiation sources have been computed with D1SUNED methodology. Activation calculations were performed with ACAB code^15^ to select the pathways leading to the formation of the 99% of radioisotopes contributing at least 99% of the contact dose rate^16^. In general, FENDL 3.1c/d^17^ was used for neutron transport, while EAF2007^18^ was used for activation and photon transport.

Both in-bioshield ITER MCNP reference models, C-Model^19^ and E-lite^8^, have been used to record the different radiation sources of In-Vessel components. The former, to record the decay gamma and photoneutron sources of the first wall panels and divertor cassette. The latter, for the torus cryopump and equatorial and upper port plugs. The complete gammas energy range was considered when computing the DGS.

The photoneutron source was computed coupling a neutron—decay photon—photoneutron simulation, where only decay photons with energy higher than 1.66 MeV (photoneutron production threshold in Be) were considered.

The radiation source of activated components remaining in-vessel was computed using SRC-UNED^7^. Information of decay photons was recorded in a cylindrical surface right behind the bioshield (r = 1470 cm) in E-lite model, and stored in an external WSSA file. To properly capture the machine configuration when this radiation source is relevant (stages 3 and 4), several modifications were implemented in E-lite. The interspace and port cell equipment, together with the bioshield plug and port plug, were removed from the geometry. D1SUNED PMT, which allow to alter the material (and density) of cells that decay photons are transported in, were used for this purpose. In this case, air “fills” in all cells belonging to the port plug, bioshield plug and interspace and port cell equipment. Photon production does neither occur in any of these cells.

The information stored in the WSSA file is then used to generate a gamma source distribution, which is used to sample and transport particles in the Tokamak Complex MCNP model. The angular extension of the generated distributions covers a single bioshield plug region. As the geometry around such areas is similar for all ports of the same component when cells are voided, only four distributions were computed. They account for: (i) upper port plug extraction at L2, (ii) equatorial port plug and first wall panels extractions at L1, (iii) divertor extraction and (iv) torus cryopump extraction, both at B1.

Calculations were performed in the Tokamak Complex MCNP model by rotating the distribution, in the azimuth angle, to match the port plug where the In-Vessel contribution is computed.

## Supplementary Information

Supplementary Information 1.

Supplementary Video 1.

## Data Availability

Data and model presented are the intellectual property of the ITER Organization. Data of the main text and the supplementary information will be made available upon reasonable request (to the corresponding author) after the recipients confirm in writing that the purpose of obtaining the data is only to reproduce the results and after the recipients have signed and returned a non-disclosure agreement confirming that no part of the data will be distributed in any way.

## Code availability

The MCNP6 v.2.0 code is distributed by the Radiation Safety Information Computational Center (RSICC, Oak Ridge National Laboratory) under user licences, following the procedure provided online (https://mcnp.lanl.gov/mcnp_how_to_get_to_mcnp.shtml). The D1SUNED v.4.1.1 code, which is developed by UNED, is a proprietary patch-code to MCNP6 v.2.0. The code will be made available on reasonable request (to the corresponding author) after the recipients confirm in writing that the purpose of obtaining the code is only to reproduce the results and after the recipients have signed and returned a non-disclosure agreement confirming that no part of the code will be distributed in any way.
