# Supplementary material for: Assessment of ITER radiation environment during the remote-handling operation of In-Vessel components with D1SUNED
Source: Sci Rep. 2023 Mar 2;13:3544. doi: 10.1038/s41598-023-30534-x (PMC9981721; doi:10.1038/s41598-023-30534-x)
Supplement: Supplementary file 1 — Supplementary Information 1. [file 41598_2023_30534_MOESM1_ESM.pdf]

# Supplementary information of

# Assessment of ITER radiation environment during the remote-handling operation of In-Vessel components with D1SUNED

P. Martínez-Albertos, P. Sauvan, M. Loughlin, Y. Le Tonqueze, R. Juárez

Supplementary Fig. **S1**.

Simplified representation of the different stages to be performed during a single cask transfer operation of ITER remote-handling maintenance scenario. Each stage mentioned in the main text comprises several substages or tasks, which are of out of the scope of this paper. As an example, several substages of stage 1 (extract the port cell equipment, bioshield plug an interspace equipment) are shown.

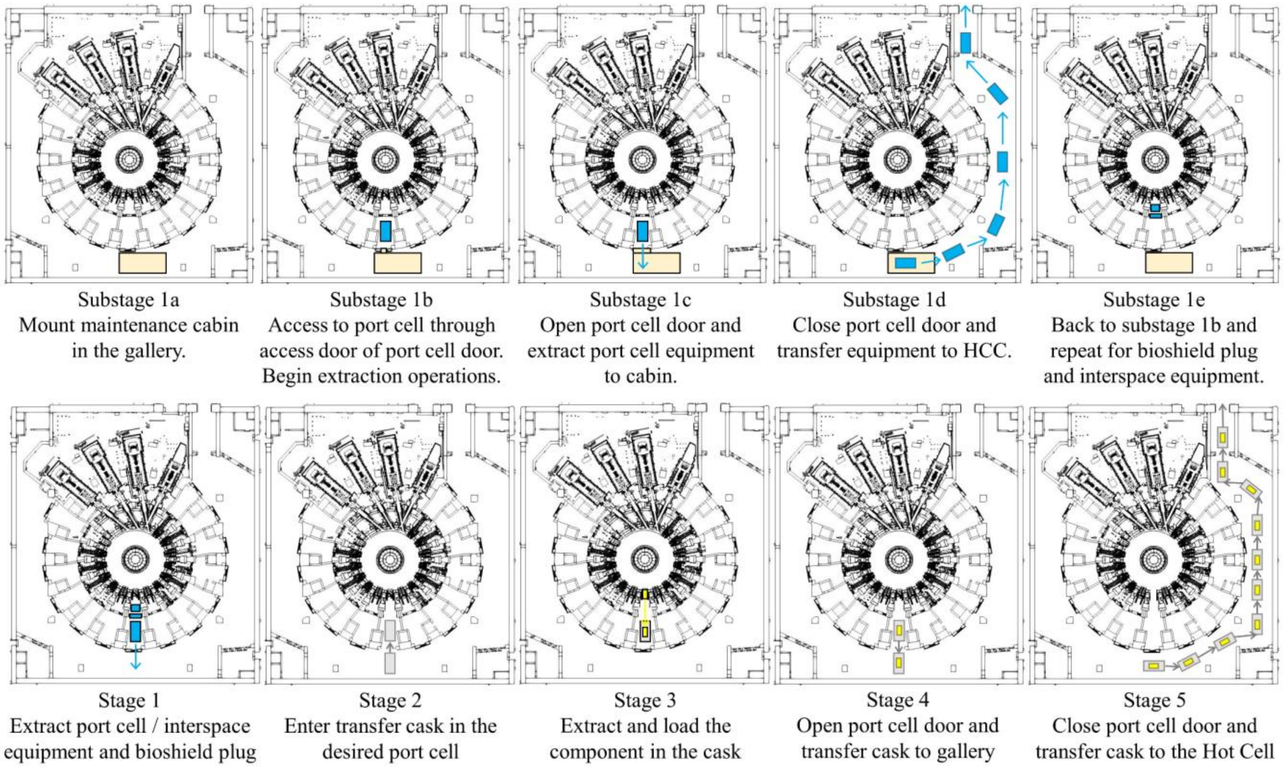

Supplementary Fig. S2.

Statistical relative errors of radiation sources considered. Left: Relative error of decay gamma sources distributions of the 5 transferred components. Right: Relative error of dose rate produced by In-Vessel components once the port cell door is open, computed with SRC-UNED methodology.

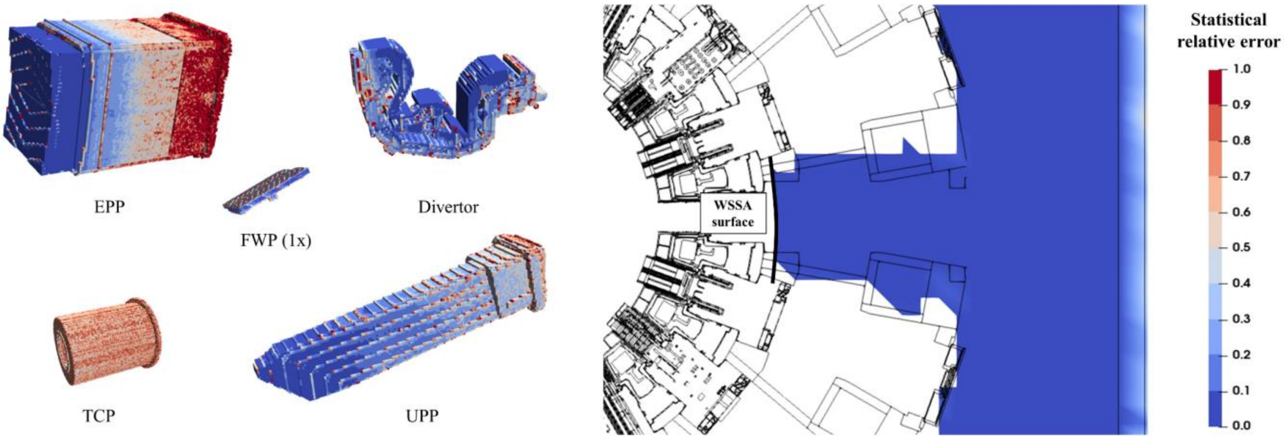

Supplementary Fig. S3.

Vertical view of the statistical relative error of the dose rate for the 3 first wall panels (left) and equatorial port plug (right) cask.

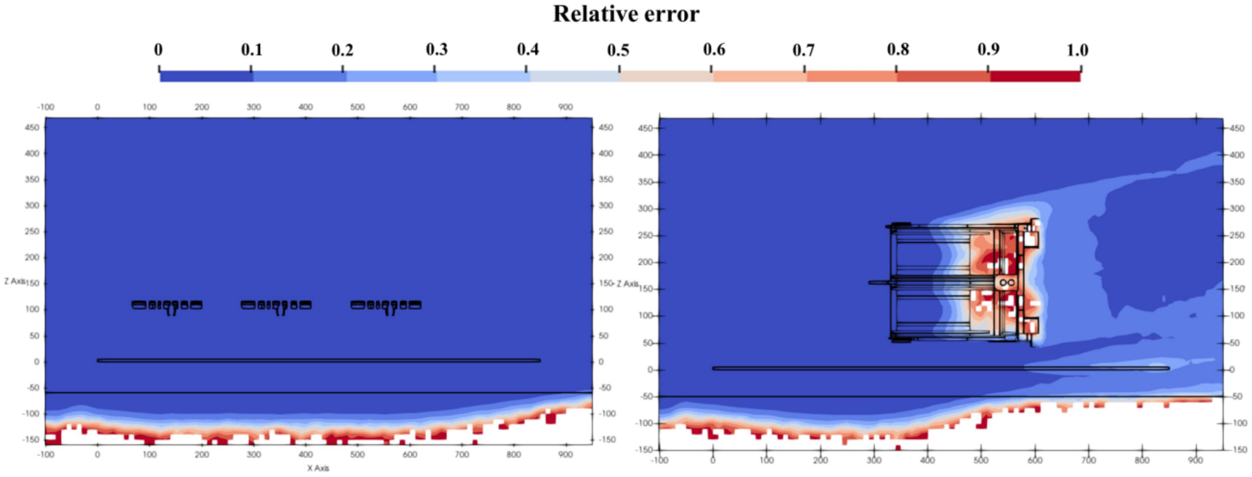

Supplementary Fig. S4.

Statistical relative error from integral total dose map produced by the extraction of 54 divertor cassettes. The shielded corners at B1 are marked.

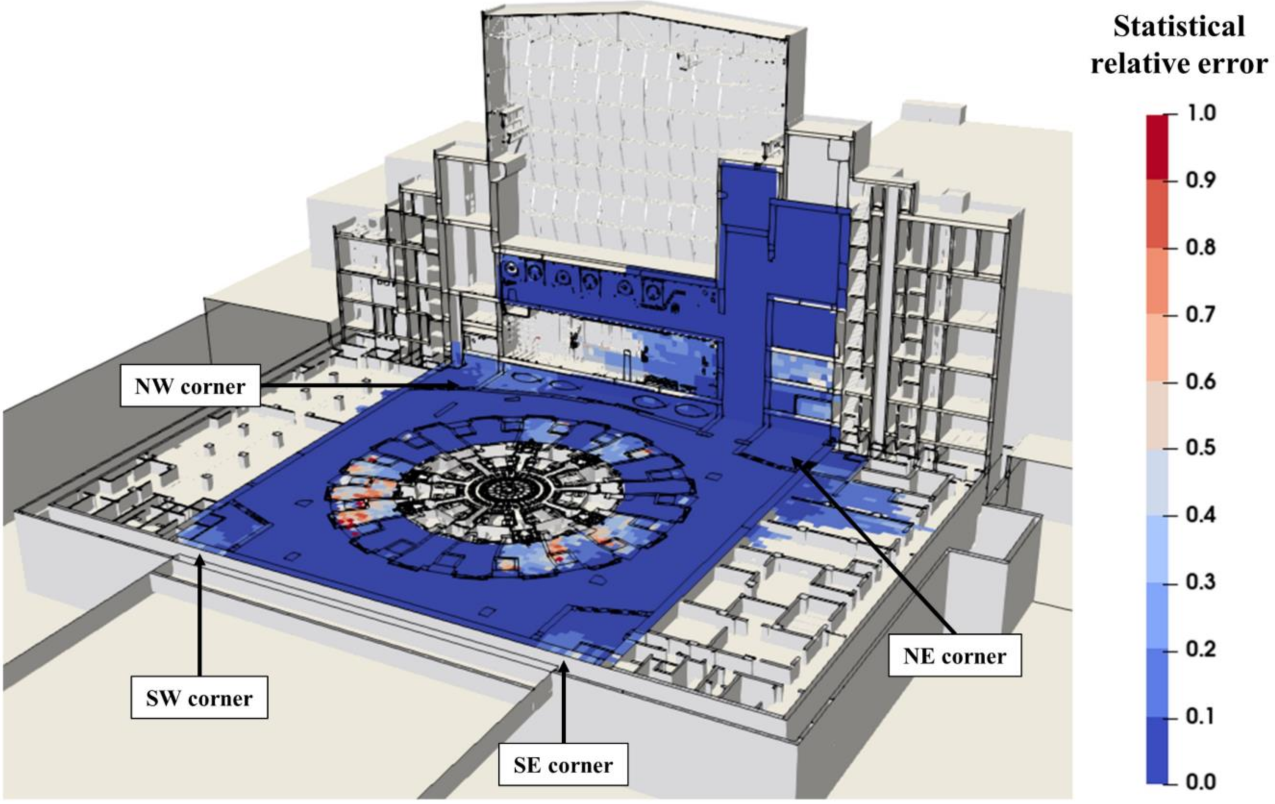

Supplementary Fig. S5.

Relative error of dose rate maps due to the cask transfer of the equatorial port plug at 16 positions along its trajectory from port cell #08 to the HCC. Relative errors of dose rates below 0.1 µSv/h are not shown.

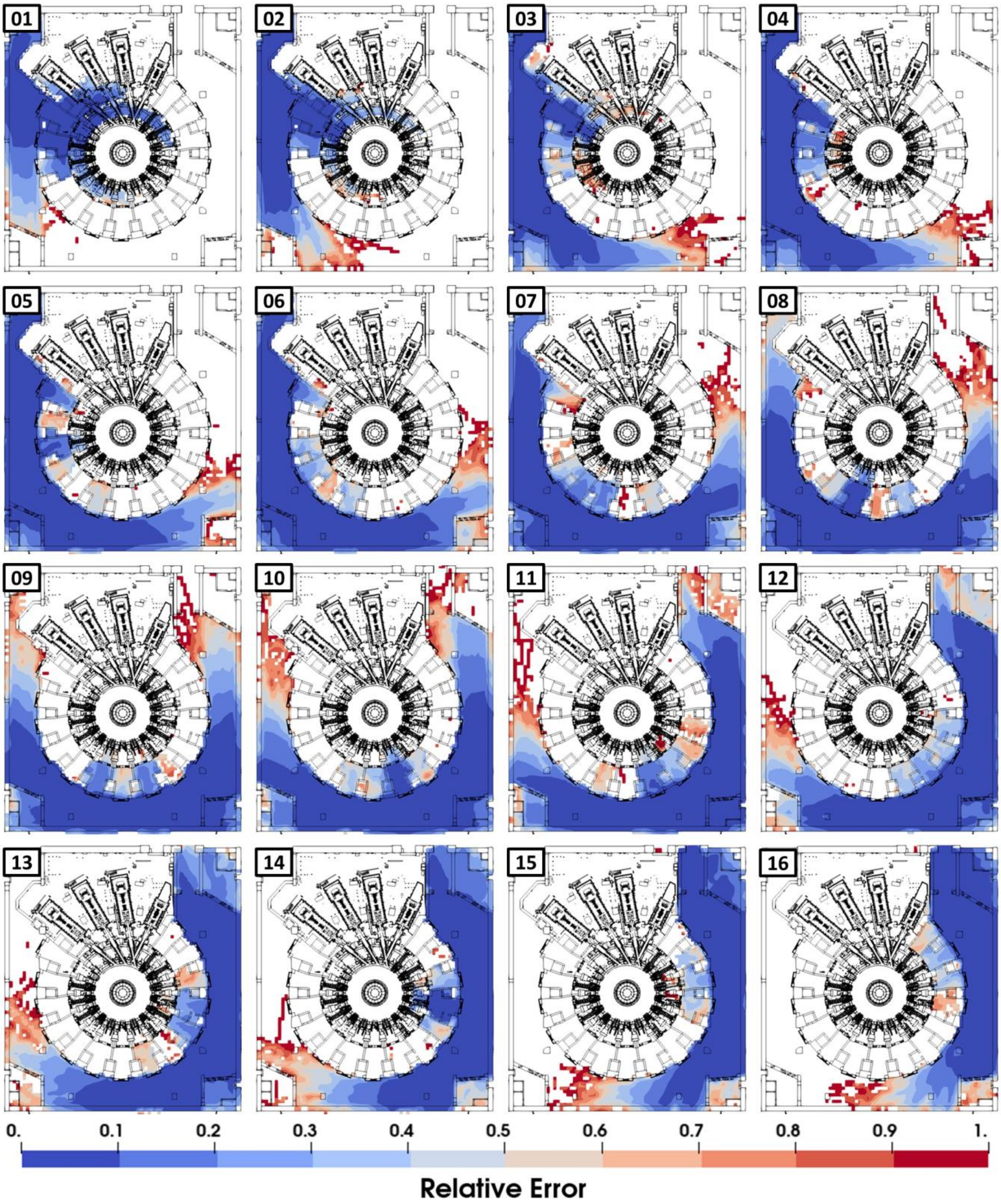

Supplementary Fig. S6.

Statistical relative errors of photoneutron flux (left), decay gamma contribution to the dose rate (centre) and photoneutron contribution to the dose rate (right) of the 3 first wall panels cask in front of the south-east shielded corner.

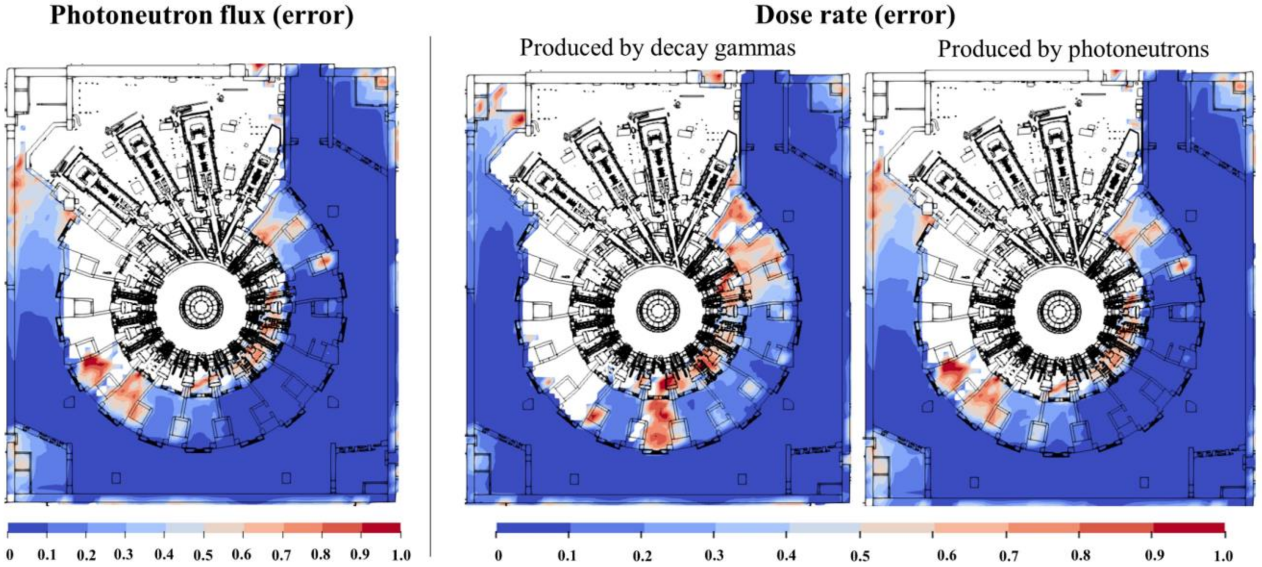

## Supplementary Table 1.

Dose rate values (in µSv/h), averaged over the shielded corners at L1 level for the transfer of the equatorial port plug and 3 first wall panels over travelled distance are given below. Coordinates of tallies used to extract dose rate values averaged on shielded corners are provided. Coordinates, in cm, are given in the following format: "Name: $x_{min}$, $x_{max}$, $y_{min}$, $y_{max}$, $z_{min}$, $z_{max}$".

- North-West shielded corner: -3475, -2511, 3445, 4305, -148, 310.
- South-West shielded corner: -3475, -2511, -3475, -2665, -148, 310.
- South-East shielded corner: 2511, 3475, -3475, -2715, -148, 310.
- North-East shielded corner: 2461, 3475, 3185, 4305, -148, 310.

| | North-West shielded corner | | South-West shielded corner | | South-East shielded corner | | North-East shielded corner | |
|---|---|---|---|---|---|---|---|---|
| **Cask travelled distance (m)** | **Port plug** | **Panels** | **Port plug** | **Panels** | **Port plug** | **Panels** | **Port plug** | **Panels** |
| 1 | 1.43E+1 | 1.18E+2 | 0.00E+0 | 7.21E-2 | 0.00E+0 | 0.00E+0 | 0.00E+0 | 0.00E+0 |
| 2 | 1.75E+1 | 1.51E+2 | 2.23E-2 | 6.62E-2 | 0.00E+0 | 0.00E+0 | 0.00E+0 | 0.00E+0 |
| 3 | 2.23E+1 | 1.90E+2 | 1.39E-2 | 6.86E-2 | 0.00E+0 | 0.00E+0 | 0.00E+0 | 0.00E+0 |
| 4 | 2.88E+1 | 2.36E+2 | 2.57E-2 | 3.30E-2 | 0.00E+0 | 0.00E+0 | 0.00E+0 | 0.00E+0 |
| 5 | 3.56E+1 | 2.98E+2 | 1.26E-2 | 1.84E-1 | 0.00E+0 | 0.00E+0 | 0.00E+0 | 0.00E+0 |
| 6 | 4.57E+1 | 3.67E+2 | 5.89E-3 | 9.30E-2 | 0.00E+0 | 0.00E+0 | 0.00E+0 | 0.00E+0 |
| 7 | 6.69E+1 | 4.57E+2 | 7.10E-3 | 3.83E-1 | 0.00E+0 | 0.00E+0 | 0.00E+0 | 0.00E+0 |
| 8 | 8.92E+1 | 5.68E+2 | 1.31E-2 | 7.27E-1 | 0.00E+0 | 0.00E+0 | 0.00E+0 | 0.00E+0 |
| 9 | 1.13E+2 | 7.17E+2 | 1.04E-1 | 2.09E+0 | 0.00E+0 | 0.00E+0 | 0.00E+0 | 0.00E+0 |
| 10 | 1.46E+2 | 9.03E+2 | 1.30E-1 | 5.46E+0 | 0.00E+0 | 0.00E+0 | 0.00E+0 | 0.00E+0 |
| 11 | 1.99E+2 | 1.15E+3 | 5.38E-1 | 6.94E+0 | 0.00E+0 | 0.00E+0 | 0.00E+0 | 0.00E+0 |
| 12 | 2.81E+2 | 1.46E+3 | 7.98E+0 | 1.34E+1 | 0.00E+0 | 0.00E+0 | 0.00E+0 | 0.00E+0 |
| 13 | 3.85E+2 | 1.93E+3 | 3.80E+1 | 1.67E+1 | 0.00E+0 | 0.00E+0 | 0.00E+0 | 0.00E+0 |
| 14 | 5.18E+2 | 2.59E+3 | 5.49E+1 | 1.97E+1 | 0.00E+0 | 0.00E+0 | 0.00E+0 | 0.00E+0 |
| 15 | 5.10E+2 | 3.25E+3 | 7.08E+1 | 2.26E+1 | 0.00E+0 | 0.00E+0 | 0.00E+0 | 0.00E+0 |
| 16 | 5.07E+2 | 4.08E+3 | 1.02E+2 | 2.79E+1 | 0.00E+0 | 0.00E+0 | 0.00E+0 | 0.00E+0 |
| 17 | 8.05E+2 | 5.32E+3 | 1.32E+2 | 2.56E+1 | 0.00E+0 | 0.00E+0 | 0.00E+0 | 0.00E+0 |
| 18 | 8.77E+2 | 5.14E+3 | 1.39E+2 | 2.46E+1 | 3.89E-4 | 0.00E+0 | 0.00E+0 | 0.00E+0 |
| 19 | 9.68E+2 | 4.57E+3 | 1.55E+2 | 2.38E+1 | 0.00E+0 | 0.00E+0 | 0.00E+0 | 0.00E+0 |
| 20 | 1.16E+3 | 4.14E+3 | 1.53E+2 | 2.19E+1 | 0.00E+0 | 0.00E+0 | 0.00E+0 | 0.00E+0 |
| 21 | 1.16E+3 | 3.82E+3 | 1.53E+2 | 2.07E+1 | 2.10E-1 | 0.00E+0 | 0.00E+0 | 0.00E+0 |
| 22 | 1.01E+3 | 3.51E+3 | 1.51E+2 | 2.38E+1 | 0.00E+0 | 0.00E+0 | 0.00E+0 | 0.00E+0 |
| 23 | 9.00E+2 | 3.24E+3 | 1.59E+2 | 2.58E+1 | 1.10E-5 | 2.70E-2 | 0.00E+0 | 0.00E+0 |
| 24 | 7.77E+2 | 3.03E+3 | 1.62E+2 | 2.58E+1 | 0.00E+0 | 3.05E-2 | 0.00E+0 | 0.00E+0 |
| 25 | 6.70E+2 | 2.81E+3 | 1.83E+2 | 2.84E+1 | 0.00E+0 | 0.00E+0 | 0.00E+0 | 0.00E+0 |
| 26 | 5.83E+2 | 2.61E+3 | 1.79E+2 | 3.24E+1 | 9.74E-2 | 0.00E+0 | 0.00E+0 | 0.00E+0 |
| 27 | 5.03E+2 | 2.46E+3 | 1.92E+2 | 3.15E+1 | 5.77E-3 | 0.00E+0 | 0.00E+0 | 0.00E+0 |
| 28 | 4.38E+2 | 2.29E+3 | 1.98E+2 | 3.50E+1 | 0.00E+0 | 0.00E+0 | 0.00E+0 | 0.00E+0 |
| 29 | 3.92E+2 | 2.17E+3 | 2.08E+2 | 3.92E+1 | 1.35E-1 | 0.00E+0 | 0.00E+0 | 0.00E+0 |
| 30 | 3.38E+2 | 2.01E+3 | 2.21E+2 | 3.72E+1 | 3.46E-1 | 2.06E-1 | 0.00E+0 | 0.00E+0 |
| 31 | 3.01E+2 | 1.90E+3 | 2.39E+2 | 3.85E+1 | 1.43E-3 | 6.25E-3 | 0.00E+0 | 0.00E+0 |
| 32 | 2.65E+2 | 1.76E+3 | 2.36E+2 | 4.41E+1 | 0.00E+0 | 3.11E-2 | 0.00E+0 | 0.00E+0 |
| 33 | 2.37E+2 | 1.68E+3 | 2.50E+2 | 5.22E+1 | 2.13E-1 | 2.98E-2 | 0.00E+0 | 0.00E+0 |
| 34 | 2.11E+2 | 1.55E+3 | 2.73E+2 | 5.17E+1 | 3.54E-1 | 2.02E-1 | 0.00E+0 | 0.00E+0 |
| 35 | 1.85E+2 | 1.47E+3 | 2.91E+2 | 5.47E+1 | 5.47E-1 | 1.06E-1 | 0.00E+0 | 0.00E+0 |
| 36 | 1.63E+2 | 1.36E+3 | 2.98E+2 | 5.85E+1 | 3.88E-1 | 0.00E+0 | 0.00E+0 | 0.00E+0 |
| 37 | 1.51E+2 | 1.28E+3 | 3.31E+2 | 6.61E+1 | 6.96E-1 | 1.33E-1 | 0.00E+0 | 0.00E+0 |
| 38 | 1.35E+2 | 1.19E+3 | 3.35E+2 | 7.11E+1 | 8.06E-1 | 2.14E-1 | 0.00E+0 | 0.00E+0 |
| 39 | 1.29E+2 | 1.11E+3 | 3.93E+2 | 7.85E+1 | 6.08E-1 | 2.55E-1 | 0.00E+0 | 0.00E+0 |
| 40 | 1.14E+2 | 1.05E+3 | 4.00E+2 | 8.77E+1 | 9.60E-1 | 1.14E-1 | 0.00E+0 | 0.00E+0 |
| 41 | 1.05E+2 | 9.70E+2 | 4.33E+2 | 9.71E+1 | 1.09E+0 | 1.19E-1 | 0.00E+0 | 0.00E+0 |
| 42 | 9.93E+1 | 9.13E+2 | 4.76E+2 | 1.08E+2 | 3.04E+0 | 8.56E-1 | 0.00E+0 | 0.00E+0 |
| 43 | 9.26E+1 | 8.69E+2 | 5.20E+2 | 1.16E+2 | 1.85E+0 | 3.40E-1 | 0.00E+0 | 0.00E+0 |
| 44 | 8.81E+1 | 8.12E+2 | 5.82E+2 | 1.38E+2 | 1.22E+0 | 5.60E-1 | 0.00E+0 | 0.00E+0 |
| 45 | 8.27E+1 | 7.80E+2 | 6.25E+2 | 1.46E+2 | 3.59E+0 | 9.16E-1 | 0.00E+0 | 0.00E+0 |
| 46 | 8.21E+1 | 7.19E+2 | 7.14E+2 | 1.75E+2 | 4.09E+0 | 8.42E-1 | 0.00E+0 | 0.00E+0 |
| 47 | 7.60E+1 | 6.87E+2 | 7.77E+2 | 1.90E+2 | 1.94E+0 | 6.80E-1 | 0.00E+0 | 0.00E+0 |
| 48 | 7.64E+1 | 6.58E+2 | 8.65E+2 | 2.27E+2 | 3.06E+0 | 7.26E-1 | 0.00E+0 | 0.00E+0 |
| 49 | 7.65E+1 | 6.19E+2 | 9.54E+2 | 2.46E+2 | 4.23E+0 | 1.65E+0 | 0.00E+0 | 0.00E+0 |
| 50 | 7.17E+1 | 5.62E+2 | 1.02E+3 | 2.93E+2 | 8.69E+0 | 2.77E+0 | 0.00E+0 | 0.00E+0 |
| 51 | 7.03E+1 | 5.21E+2 | 1.09E+3 | 3.24E+2 | 1.27E+1 | 2.96E+0 | 0.00E+0 | 0.00E+0 |
| 52 | 9.05E+1 | 5.24E+2 | 1.22E+3 | 3.82E+2 | 3.11E+1 | 4.49E+0 | 0.00E+0 | 0.00E+0 |
| 53 | 1.50E+2 | 5.39E+2 | 1.35E+3 | 4.93E+2 | 3.40E+1 | 5.96E+0 | 0.00E+0 | 0.00E+0 |
| 54 | 1.86E+2 | 5.47E+2 | 1.75E+3 | 5.76E+2 | 4.41E+1 | 1.25E+1 | 0.00E+0 | 0.00E+0 |
| 55 | 1.95E+2 | 5.30E+2 | 1.85E+3 | 5.57E+2 | 6.59E+1 | 1.23E+1 | 0.00E+0 | 0.00E+0 |
| 56 | 1.95E+2 | 5.00E+2 | 1.06E+3 | 5.86E+2 | 8.44E+1 | 1.35E+1 | 0.00E+0 | 0.00E+0 |
| 57 | 1.60E+2 | 3.96E+2 | 6.47E+2 | 6.01E+2 | 9.41E+1 | 1.42E+1 | 0.00E+0 | 0.00E+0 |
| 58 | 2.78E+1 | 3.08E+2 | 6.52E+2 | 6.11E+2 | 1.01E+2 | 1.78E+1 | 0.00E+0 | 0.00E+0 |
| 59 | 1.73E+1 | 2.18E+2 | 7.25E+2 | 5.14E+2 | 1.17E+2 | 2.19E+1 | 0.00E+0 | 0.00E+0 |
| 60 | 1.07E+1 | 1.37E+2 | 7.14E+2 | 4.81E+2 | 1.31E+2 | 2.17E+1 | 0.00E+0 | 0.00E+0 |
| 61 | 7.18E+0 | 7.69E+1 | 6.58E+2 | 4.65E+2 | 1.41E+2 | 2.44E+1 | 6.57E-6 | 0.00E+0 |
| 62 | 4.80E+0 | 3.20E+1 | 7.07E+2 | 4.67E+2 | 1.69E+2 | 3.25E+1 | 0.00E+0 | 0.00E+0 |

| | | | | | | | | |
|---|---|---|---|---|---|---|---|---|
| 63 | 4.84E+0 | 2.20E+1 | 7.95E+2 | 4.36E+2 | 1.89E+2 | 3.22E+1 | 0.00E+0 | 0.00E+0 |
| 64 | 3.27E+0 | 1.68E+1 | 8.53E+2 | 4.46E+2 | 2.09E+2 | 4.56E+1 | 0.00E+0 | 0.00E+0 |
| 65 | 1.73E+0 | 1.31E+1 | 8.79E+2 | 5.32E+2 | 2.24E+2 | 4.79E+1 | 0.00E+0 | 0.00E+0 |
| 66 | 1.79E+0 | 1.03E+1 | 9.09E+2 | 6.30E+2 | 2.55E+2 | 5.16E+1 | 0.00E+0 | 0.00E+0 |
| 67 | 1.68E+0 | 8.90E+0 | 9.74E+2 | 7.79E+2 | 2.59E+2 | 5.83E+1 | 0.00E+0 | 0.00E+0 |
| 68 | 1.28E+0 | 7.84E+0 | 1.03E+3 | 9.23E+2 | 3.19E+2 | 6.80E+1 | 0.00E+0 | 0.00E+0 |
| 69 | 1.33E+0 | 5.90E+0 | 1.08E+3 | 1.09E+3 | 3.83E+2 | 7.63E+1 | 0.00E+0 | 0.00E+0 |
| 70 | 1.17E+0 | 4.34E+0 | 1.09E+3 | 1.21E+3 | 4.78E+2 | 8.33E+1 | 0.00E+0 | 0.00E+0 |
| 71 | 6.52E-1 | 3.99E+0 | 1.07E+3 | 1.24E+3 | 5.73E+2 | 1.13E+2 | 0.00E+0 | 0.00E+0 |
| 72 | 6.62E-1 | 3.96E+0 | 9.33E+2 | 1.14E+3 | 7.40E+2 | 1.37E+2 | 0.00E+0 | 0.00E+0 |
| 73 | 4.23E-1 | 2.92E+0 | 3.24E+2 | 1.01E+3 | 9.25E+2 | 1.54E+2 | 0.00E+0 | 0.00E+0 |
| 74 | 6.16E-1 | 1.85E+0 | 2.14E+2 | 8.48E+2 | 1.13E+3 | 1.69E+2 | 0.00E+0 | 0.00E+0 |
| 75 | 3.97E-1 | 1.99E+0 | 2.33E+2 | 6.85E+2 | 1.31E+3 | 1.94E+2 | 0.00E+0 | 0.00E+0 |
| 76 | 2.09E-1 | 1.98E+0 | 2.15E+2 | 5.20E+2 | 1.53E+3 | 2.34E+2 | 4.84E-2 | 5.83E-2 |
| 77 | 1.77E-1 | 1.31E+0 | 2.21E+2 | 3.80E+2 | 1.71E+3 | 2.54E+2 | 1.00E-2 | 0.00E+0 |
| 78 | 3.95E-1 | 1.06E+0 | 2.13E+2 | 3.42E+2 | 1.97E+3 | 2.82E+2 | 0.00E+0 | 0.00E+0 |
| 79 | 1.01E-1 | 9.67E-1 | 2.09E+2 | 3.53E+2 | 2.03E+3 | 3.00E+2 | 0.00E+0 | 0.00E+0 |
| 80 | 2.09E-1 | 8.67E-1 | 2.08E+2 | 3.54E+2 | 2.12E+3 | 3.16E+2 | 2.16E-1 | 0.00E+0 |
| 81 | 5.44E-2 | 8.86E-1 | 1.89E+2 | 3.64E+2 | 2.19E+3 | 3.47E+2 | 1.67E-1 | 0.00E+0 |
| 82 | 1.08E-1 | 3.97E-1 | 2.04E+2 | 3.56E+2 | 2.23E+3 | 3.39E+2 | 8.28E-1 | 0.00E+0 |
| 83 | 5.38E-3 | 3.55E-1 | 2.04E+2 | 3.66E+2 | 2.13E+3 | 3.83E+2 | 6.17E-1 | 0.00E+0 |
| 84 | 2.67E-1 | 3.35E-1 | 1.85E+2 | 3.55E+2 | 1.95E+3 | 4.06E+2 | 6.65E-1 | 0.00E+0 |
| 85 | 4.26E-2 | 4.56E-1 | 1.50E+2 | 3.49E+2 | 1.82E+3 | 3.92E+2 | 5.80E-1 | 1.39E-1 |
| 86 | 4.77E-2 | 3.78E-1 | 1.28E+2 | 3.63E+2 | 1.64E+3 | 4.02E+2 | 8.45E-6 | 2.39E-2 |
| 87 | 4.75E-2 | 3.93E-1 | 1.16E+2 | 3.78E+2 | 1.51E+3 | 4.09E+2 | 2.45E-2 | 1.91E-1 |
| 88 | 5.43E-2 | 3.35E-1 | 9.98E+1 | 3.86E+2 | 1.46E+3 | 4.62E+2 | 5.54E-1 | 6.36E-2 |
| 89 | 1.29E-1 | 3.96E-1 | 8.06E+1 | 3.84E+2 | 1.38E+3 | 4.47E+2 | 6.50E-1 | 0.00E+0 |
| 90 | 1.41E-1 | 3.24E-1 | 6.23E+1 | 3.67E+2 | 1.31E+3 | 4.69E+2 | 9.24E-1 | 8.58E-2 |
| 91 | 2.10E-2 | 2.04E-1 | 5.26E+1 | 3.43E+2 | 1.22E+3 | 4.79E+2 | 3.65E-1 | 6.70E-2 |
| 92 | 4.45E-2 | 2.15E-1 | 5.09E+1 | 3.13E+2 | 1.09E+3 | 4.80E+2 | 1.11E+0 | 2.97E-2 |
| 93 | 3.14E-2 | 8.95E-2 | 4.64E+1 | 3.02E+2 | 1.02E+3 | 5.12E+2 | 9.96E-1 | 6.48E-2 |
| 94 | 2.98E-2 | 8.29E-2 | 4.45E+1 | 2.78E+2 | 1.18E+3 | 6.02E+2 | 8.23E-1 | 6.27E-1 |
| 95 | 1.00E-2 | 5.75E-2 | 5.58E+1 | 2.46E+2 | 1.64E+3 | 7.77E+2 | 1.53E+0 | 2.10E-1 |
| 96 | 0.00E+0 | 1.41E-1 | 4.68E+1 | 2.35E+2 | 2.84E+3 | 1.06E+3 | 6.71E-1 | 3.87E-1 |
| 97 | 0.00E+0 | 8.74E-2 | 4.62E+1 | 2.24E+2 | 5.35E+3 | 1.35E+3 | 1.18E+0 | 1.54E-3 |
| 98 | 0.00E+0 | 9.67E-2 | 4.68E+1 | 2.15E+2 | 7.91E+3 | 1.69E+3 | 1.84E+0 | 2.34E-1 |
| 99 | 1.58E-2 | 3.08E-2 | 4.09E+1 | 2.05E+2 | 9.19E+3 | 1.97E+3 | 2.29E+0 | 5.89E-1 |
| 100 | 3.06E-3 | 3.91E-2 | 2.72E+1 | 1.79E+2 | 8.76E+3 | 2.24E+3 | 3.25E+0 | 4.79E-1 |
| 101 | 0.00E+0 | 2.71E-2 | 2.25E+1 | 1.69E+2 | 7.39E+3 | 2.37E+3 | 4.01E+0 | 4.79E-2 |
| 102 | 0.00E+0 | 1.31E-2 | 2.01E+1 | 1.40E+2 | 4.74E+3 | 2.16E+3 | 4.40E+0 | 1.45E+0 |
| 103 | 0.00E+0 | 0.00E+0 | 2.24E+1 | 1.34E+2 | 2.52E+3 | 1.87E+3 | 5.44E+0 | 8.82E-1 |
| 104 | 2.32E-2 | 3.58E-5 | 2.26E+1 | 1.21E+2 | 1.25E+3 | 1.55E+3 | 4.07E+0 | 1.90E+0 |
| 105 | 0.00E+0 | 0.00E+0 | 2.31E+1 | 8.90E+1 | 7.70E+2 | 1.23E+3 | 7.76E+0 | 9.83E-1 |
| 106 | 0.00E+0 | 0.00E+0 | 1.64E+1 | 6.88E+1 | 5.27E+2 | 9.62E+2 | 5.25E+0 | 2.39E+0 |
| 107 | 0.00E+0 | 3.97E-2 | 1.77E+1 | 5.50E+1 | 3.86E+2 | 7.40E+2 | 6.44E+0 | 1.25E+0 |
| 108 | 0.00E+0 | 3.02E-2 | 1.16E+1 | 4.60E+1 | 3.03E+2 | 6.05E+2 | 1.20E+1 | 4.71E+0 |
| 109 | 0.00E+0 | 0.00E+0 | 9.18E+0 | 4.00E+1 | 2.86E+2 | 5.86E+2 | 1.68E+1 | 5.35E+0 |
| 110 | 0.00E+0 | 1.57E-2 | 6.69E+0 | 2.76E+1 | 3.94E+2 | 7.22E+2 | 1.68E+1 | 6.64E+0 |
| 111 | 0.00E+0 | 0.00E+0 | 4.75E+0 | 2.95E+1 | 6.36E+2 | 1.02E+3 | 3.22E+1 | 2.66E+1 |
| 112 | 0.00E+0 | 1.40E-2 | 4.44E+0 | 2.25E+1 | 1.21E+3 | 1.35E+3 | 6.95E+1 | 4.92E+1 |
| 113 | 0.00E+0 | 0.00E+0 | 2.99E+0 | 1.78E+1 | 1.62E+3 | 1.72E+3 | 3.85E+2 | 8.00E+1 |
| 114 | 0.00E+0 | 0.00E+0 | 2.98E+0 | 1.77E+1 | 1.64E+3 | 2.05E+3 | 5.99E+2 | 1.16E+2 |
| 115 | 0.00E+0 | 0.00E+0 | 3.70E+0 | 1.24E+1 | 1.43E+3 | 2.30E+3 | 6.20E+2 | 1.55E+2 |
| 116 | 0.00E+0 | 0.00E+0 | 2.08E+0 | 1.02E+1 | 1.29E+3 | 2.48E+3 | 6.17E+2 | 1.65E+2 |
| 117 | 0.00E+0 | 0.00E+0 | 1.02E+0 | 1.20E+1 | 1.09E+3 | 2.47E+3 | 6.86E+2 | 2.08E+2 |
| 118 | 0.00E+0 | 0.00E+0 | 1.68E+0 | 9.90E+0 | 8.03E+2 | 2.23E+3 | 7.77E+2 | 1.95E+2 |
| 119 | 0.00E+0 | 3.52E-2 | 3.08E+0 | 1.11E+1 | 4.55E+2 | 1.96E+3 | 9.57E+2 | 1.79E+2 |
| 120 | 0.00E+0 | 0.00E+0 | 2.14E+0 | 8.54E+0 | 2.53E+2 | 1.72E+3 | 1.03E+3 | 1.73E+2 |
| 121 | 0.00E+0 | 0.00E+0 | 8.74E-1 | 5.60E+0 | 1.87E+2 | 1.54E+3 | 1.05E+3 | 1.65E+2 |
| 122 | 0.00E+0 | 0.00E+0 | 4.28E-1 | 4.78E+0 | 1.34E+2 | 1.25E+3 | 1.10E+3 | 1.78E+2 |
| 123 | 0.00E+0 | 0.00E+0 | 1.72E-1 | 2.92E+0 | 8.06E+1 | 1.04E+3 | 1.12E+3 | 1.78E+2 |
| 124 | 0.00E+0 | 0.00E+0 | 3.37E-1 | 1.63E+0 | 5.16E+1 | 8.92E+2 | 1.17E+3 | 1.75E+2 |
| 125 | 0.00E+0 | 0.00E+0 | 1.13E-1 | 1.15E+0 | 3.83E+1 | 7.63E+2 | 1.32E+3 | 1.99E+2 |
| 126 | 0.00E+0 | 0.00E+0 | 2.72E-1 | 1.23E+0 | 3.43E+1 | 6.32E+2 | 1.21E+3 | 1.80E+2 |
| 127 | 0.00E+0 | 0.00E+0 | 7.13E-1 | 6.34E-1 | 2.93E+1 | 5.44E+2 | 1.40E+3 | 2.02E+2 |
| 128 | 0.00E+0 | 0.00E+0 | 9.98E-1 | 4.81E-1 | 2.48E+1 | 4.72E+2 | 1.49E+3 | 2.31E+2 |
| 129 | 0.00E+0 | 0.00E+0 | 0.00E+0 | 3.09E-1 | 2.04E+1 | 4.02E+2 | 1.50E+3 | 2.28E+2 |
| 130 | 0.00E+0 | 0.00E+0 | 3.25E-1 | 1.53E+0 | 1.74E+1 | 3.57E+2 | 1.58E+3 | 2.39E+2 |
| 131 | 0.00E+0 | 0.00E+0 | 6.83E-1 | 3.22E-1 | 1.57E+1 | 3.11E+2 | 1.70E+3 | 2.44E+2 |
| 132 | 0.00E+0 | 0.00E+0 | 1.09E-2 | 3.25E-1 | 1.40E+1 | 2.64E+2 | 1.79E+3 | 2.84E+2 |
| 133 | 0.00E+0 | 0.00E+0 | 8.17E-1 | 4.34E-1 | 1.47E+1 | 2.41E+2 | 1.84E+3 | 3.03E+2 |
| 134 | 0.00E+0 | 0.00E+0 | 0.00E+0 | 1.67E-1 | 1.00E+1 | 2.16E+2 | 1.98E+3 | 3.18E+2 |
| 135 | 0.00E+0 | 0.00E+0 | 0.00E+0 | 3.65E-1 | 9.88E+0 | 1.89E+2 | 2.08E+3 | 3.46E+2 |
| 136 | 0.00E+0 | 0.00E+0 | 6.36E-2 | 6.13E-2 | 8.07E+0 | 1.70E+2 | 2.26E+3 | 3.68E+2 |
| 137 | 0.00E+0 | 0.00E+0 | 2.38E-2 | 5.74E-2 | 7.59E+0 | 1.52E+2 | 2.38E+3 | 4.18E+2 |
| 138 | 0.00E+0 | 0.00E+0 | 0.00E+0 | 6.93E-2 | 6.66E+0 | 1.42E+2 | 2.56E+3 | 4.31E+2 |
| 139 | 0.00E+0 | 0.00E+0 | 0.00E+0 | 1.63E-2 | 5.98E+0 | 1.24E+2 | 2.66E+3 | 4.76E+2 |

| | | | | | | | | |
|---|---|---|---|---|---|---|---|---|
| 140 | 0.00E+0 | 0.00E+0 | 0.00E+0 | 3.23E-3 | 5.57E+0 | 1.20E+2 | 2.85E+3 | 5.03E+2 |
| 141 | 0.00E+0 | 0.00E+0 | 0.00E+0 | 0.00E+0 | 7.15E+0 | 1.05E+2 | 2.98E+3 | 5.46E+2 |
| 142 | 0.00E+0 | 0.00E+0 | 0.00E+0 | 3.88E-2 | 5.53E+0 | 9.41E+1 | 3.20E+3 | 5.83E+2 |
| 143 | 0.00E+0 | 0.00E+0 | 0.00E+0 | 0.00E+0 | 5.08E+0 | 8.53E+1 | 3.46E+3 | 6.30E+2 |
| 144 | 0.00E+0 | 0.00E+0 | 0.00E+0 | 0.00E+0 | 4.83E+0 | 8.35E+1 | 3.70E+3 | 6.85E+2 |
| 145 | 0.00E+0 | 0.00E+0 | 3.75E-2 | 2.83E-3 | 3.34E+0 | 7.21E+1 | 4.06E+3 | 7.09E+2 |
| 146 | 0.00E+0 | 0.00E+0 | 0.00E+0 | 0.00E+0 | 2.85E+0 | 7.10E+1 | 4.20E+3 | 7.89E+2 |
| 147 | 0.00E+0 | 0.00E+0 | 0.00E+0 | 0.00E+0 | 3.32E+0 | 6.39E+1 | 4.61E+3 | 8.64E+2 |
| 148 | 0.00E+0 | 0.00E+0 | 0.00E+0 | 0.00E+0 | 5.12E+0 | 6.17E+1 | 5.19E+3 | 9.25E+2 |
| 149 | 0.00E+0 | 0.00E+0 | 0.00E+0 | 0.00E+0 | 7.53E+0 | 6.31E+1 | 5.83E+3 | 1.06E+3 |
| 150 | 0.00E+0 | 0.00E+0 | 0.00E+0 | 0.00E+0 | 6.02E+0 | 5.14E+1 | 6.59E+3 | 1.19E+3 |
| 151 | 0.00E+0 | 0.00E+0 | 0.00E+0 | 0.00E+0 | 5.81E+0 | 5.08E+1 | 7.35E+3 | 1.33E+3 |
| 152 | 0.00E+0 | 0.00E+0 | 0.00E+0 | 1.14E-2 | 5.91E+0 | 4.94E+1 | 8.13E+3 | 1.50E+3 |
| 153 | 0.00E+0 | 0.00E+0 | 0.00E+0 | 0.00E+0 | 3.18E+0 | 4.46E+1 | 9.11E+3 | 1.63E+3 |
| 154 | 0.00E+0 | 0.00E+0 | 0.00E+0 | 0.00E+0 | 5.09E+0 | 4.17E+1 | 1.01E+4 | 1.84E+3 |
| 155 | 0.00E+0 | 0.00E+0 | 0.00E+0 | 0.00E+0 | 4.12E+0 | 3.89E+1 | 1.12E+4 | 1.98E+3 |
| 156 | 0.00E+0 | 0.00E+0 | 0.00E+0 | 0.00E+0 | 5.72E+0 | 3.49E+1 | 1.22E+4 | 2.17E+3 |
| 157 | 0.00E+0 | 0.00E+0 | 0.00E+0 | 0.00E+0 | 5.88E+0 | 3.72E+1 | 1.23E+4 | 2.23E+3 |
| 158 | 0.00E+0 | 0.00E+0 | 0.00E+0 | 0.00E+0 | 8.19E+0 | 3.92E+1 | 1.06E+4 | 2.24E+3 |
| 159 | 0.00E+0 | 0.00E+0 | 0.00E+0 | 0.00E+0 | 7.65E+0 | 3.53E+1 | 7.41E+3 | 2.08E+3 |
| 160 | 0.00E+0 | 1.58E-8 | 0.00E+0 | 0.00E+0 | 4.88E+0 | 2.56E+1 | 3.82E+3 | 1.82E+3 |
| 161 | 0.00E+0 | 0.00E+0 | 0.00E+0 | 0.00E+0 | 3.68E-1 | 2.03E+1 | 1.49E+3 | 1.53E+3 |
| 162 | 0.00E+0 | 0.00E+0 | 0.00E+0 | 0.00E+0 | 1.21E-1 | 1.26E+1 | 7.40E+2 | 1.25E+3 |
| 163 | 0.00E+0 | 0.00E+0 | 0.00E+0 | 0.00E+0 | 8.30E-2 | 7.35E+0 | 4.41E+2 | 9.87E+2 |
| 164 | 0.00E+0 | 0.00E+0 | 0.00E+0 | 0.00E+0 | 7.94E-2 | 1.13E+0 | 2.67E+2 | 7.46E+2 |
| 165 | 0.00E+0 | 0.00E+0 | 0.00E+0 | 0.00E+0 | 7.37E-1 | 4.01E-1 | 1.72E+2 | 5.50E+2 |
| 166 | 0.00E+0 | 0.00E+0 | 0.00E+0 | 0.00E+0 | 3.56E-2 | 1.94E-1 | 1.42E+2 | 4.07E+2 |
| 167 | 0.00E+0 | 0.00E+0 | 0.00E+0 | 0.00E+0 | 5.75E-2 | 1.89E-1 | 1.83E+2 | 3.04E+2 |
| 168 | 0.00E+0 | 0.00E+0 | 0.00E+0 | 0.00E+0 | 1.12E-1 | 3.40E-1 | 2.34E+2 | 2.27E+2 |
| 169 | 0.00E+0 | 0.00E+0 | 0.00E+0 | 0.00E+0 | 1.84E-1 | 2.76E-1 | 2.71E+2 | 2.19E+2 |
| 170 | 0.00E+0 | 0.00E+0 | 0.00E+0 | 0.00E+0 | 1.50E-1 | 4.52E-1 | 3.85E+2 | 2.74E+2 |
| 171 | 0.00E+0 | 1.24E-8 | 0.00E+0 | 0.00E+0 | 1.36E-1 | 4.32E-1 | 7.06E+2 | 4.35E+2 |
| 172 | 0.00E+0 | 1.54E-9 | 0.00E+0 | 0.00E+0 | 8.06E-2 | 4.22E-1 | 1.35E+3 | 6.69E+2 |
| 173 | 6.84E-10 | 4.41E-8 | 0.00E+0 | 0.00E+0 | 5.08E-2 | 4.24E-1 | 2.07E+3 | 9.74E+2 |
| 174 | 5.62E-8 | 1.28E-7 | 0.00E+0 | 0.00E+0 | 6.41E-2 | 4.32E-1 | 2.42E+3 | 1.31E+3 |
| 175 | 1.75E-7 | 2.47E-7 | 0.00E+0 | 0.00E+0 | 2.76E-2 | 1.52E-1 | 2.64E+3 | 1.66E+3 |
| 176 | 3.23E-7 | 2.26E-7 | 0.00E+0 | 0.00E+0 | 1.13E-3 | 1.07E-1 | 2.73E+3 | 1.99E+3 |
| 177 | 7.28E-7 | 4.01E-7 | 0.00E+0 | 0.00E+0 | 4.35E-4 | 7.79E-2 | 2.74E+3 | 2.23E+3 |
| 178 | 2.25E-7 | 2.00E-7 | 0.00E+0 | 0.00E+0 | 0.00E+0 | 1.91E-1 | 2.70E+3 | 2.39E+3 |
| 179 | 4.30E-7 | 5.63E-7 | 0.00E+0 | 0.00E+0 | 1.08E-3 | 9.88E-2 | 2.62E+3 | 2.47E+3 |
| 180 | 4.33E-7 | 5.35E-7 | 0.00E+0 | 0.00E+0 | 0.00E+0 | 1.05E-2 | 2.48E+3 | 2.48E+3 |
| 181 | 3.29E-7 | 6.14E-7 | 0.00E+0 | 0.00E+0 | 6.11E-4 | 1.67E-2 | 2.24E+3 | 2.41E+3 |
| 182 | 4.54E-7 | 6.10E-7 | 0.00E+0 | 0.00E+0 | 0.00E+0 | 2.47E-3 | 2.09E+3 | 2.36E+3 |
| 183 | 2.76E-7 | 1.10E-6 | 0.00E+0 | 0.00E+0 | 0.00E+0 | 6.00E-3 | 2.08E+3 | 2.36E+3 |
| 184 | 2.65E-7 | 1.37E-6 | 0.00E+0 | 0.00E+0 | 3.05E-4 | 7.49E-4 | 2.01E+3 | 2.33E+3 |
| 185 | 1.83E-7 | 2.55E-7 | 0.00E+0 | 0.00E+0 | 6.82E-4 | 7.76E-5 | 1.61E+3 | 2.14E+3 |
| 186 | 2.78E-7 | 3.65E-7 | 0.00E+0 | 0.00E+0 | 0.00E+0 | 8.41E-3 | 1.10E+3 | 1.82E+3 |
| 187 | 5.28E-8 | 1.71E-7 | 0.00E+0 | 0.00E+0 | 2.87E-3 | 1.78E-4 | 6.18E+2 | 1.46E+3 |
| 188 | 5.51E-9 | 2.61E-7 | 0.00E+0 | 0.00E+0 | 1.72E-4 | 0.00E+0 | 1.73E+2 | 1.10E+3 |
| 189 | 0.00E+0 | 1.09E-7 | 0.00E+0 | 0.00E+0 | 0.00E+0 | 0.00E+0 | 3.63E+1 | 7.51E+2 |
| 190 | 0.00E+0 | 1.58E-9 | 0.00E+0 | 0.00E+0 | 0.00E+0 | 0.00E+0 | 1.11E+1 | 4.49E+2 |
| 191 | 0.00E+0 | 0.00E+0 | 0.00E+0 | 0.00E+0 | 2.75E-5 | 0.00E+0 | 4.81E+0 | 2.15E+2 |
| 192 | 0.00E+0 | 0.00E+0 | 0.00E+0 | 0.00E+0 | 0.00E+0 | 4.62E-3 | 2.43E+0 | 9.47E+1 |
| 193 | 0.00E+0 | 0.00E+0 | 0.00E+0 | 0.00E+0 | 0.00E+0 | 0.00E+0 | 1.24E+0 | 4.46E+1 |
| 194 | 0.00E+0 | 0.00E+0 | 0.00E+0 | 0.00E+0 | 0.00E+0 | 0.00E+0 | 6.89E-1 | 2.32E+1 |
| 195 | 0.00E+0 | 0.00E+0 | 0.00E+0 | 0.00E+0 | 0.00E+0 | 2.10E-3 | 3.95E-1 | 1.33E+1 |
| 196 | 0.00E+0 | 0.00E+0 | 0.00E+0 | 0.00E+0 | 0.00E+0 | 0.00E+0 | 3.49E-1 | 8.34E+0 |
| 197 | 0.00E+0 | 0.00E+0 | 0.00E+0 | 0.00E+0 | 0.00E+0 | 0.00E+0 | 1.92E-1 | 5.43E+0 |
| 198 | 0.00E+0 | 0.00E+0 | 0.00E+0 | 0.00E+0 | 0.00E+0 | 0.00E+0 | 1.60E-1 | 3.74E+0 |
| 199 | 0.00E+0 | 0.00E+0 | 0.00E+0 | 0.00E+0 | 0.00E+0 | 1.23E-4 | 5.74E-2 | 1.10E+0 |
